# Molecular mechanism of azoxy bond formation for azoxymycins biosynthesis

**DOI:** 10.1038/s41467-019-12250-1

**Published:** 2019-10-08

**Authors:** Yuan-Yang Guo, Zhen-Hua Li, Tian-Yu Xia, Yi-Ling Du, Xu-Ming Mao, Yong-Quan Li

**Affiliations:** 1 Institute of Pharmaceutical Biotechnology & First Affiliated Hospital, Zhejiang University School of Medicine, 310058 Hangzhou, China; 2 School of Chemistry and Chemical Engineering, Henan Normal University, 453007 Xinxiang, China

**Keywords:** Biochemistry, Biocatalysis, Biosynthesis

## Abstract

Azoxy bond is an important chemical bond and plays a crucial role in high energy density materials. However, the biosynthetic mechanism of azoxy bond remains enigmatic. Here we report that the azoxy bond biosynthesis of azoxymycins is an enzymatic and non-enzymatic coupling cascade reaction. In the first step, nonheme diiron *N*-oxygenase AzoC catalyzes the oxidization of amine to its nitroso analogue. Redox coenzyme pairs then facilitate the mutual conversion between nitroso group and hydroxylamine via the radical transient intermediates, which efficiently dimerize to azoxy bond. The deficiency of nucleophilic reactivity in AzoC is proposed to account for the enzyme’s non-canonical oxidization of amine to nitroso product. Free nitrogen radicals induced by coenzyme pairs are proposed to be responsible for the efficient non-enzymatic azoxy bond formation. This mechanism study will provide molecular basis for the biosynthesis of azoxy high energy density materials and other valuable azoxy chemicals.

## Abstract (web summary)

Azoxy bonds are frequently found in liquid crystals, chemical intermediates, dyes, agrochemicals and pharmaceuticals. Here, the authors investigated azoxy bond formation by the non-heme diiron N-oxygenase AzoC in azoxymycin biosynthesis and show that the nitroso group plays a key part in it.

## Introduction

Azoxy bond, also known as diazene oxide, is an important chemical bond in liquid crystals, chemical intermediates, dyes, agrochemicals, and pharmaceuticals^1^. Especially, it plays an important role in the synthesis of high-energy density materials (HEDM), as it can significantly improve the materials’ energy level by increasing their physical density and energy strength while concurrently decreasing their sensitivity for a better storage safety^2^. However, the chemical synthesis of these types of compounds is challenged by various problems, such as the low synthetic efficiency, uncontrollable stereo and spatial selectivity, poor atom economy, and risky issue associated with synthetic safety. In overcoming these issues, the efficient biosynthesis of these promising compounds would clearly be of high benefit to both the chemical industry and the academia^3^. To date, although a few azoxy natural products have been reported (Supplementary Fig. 1), the biosynthetic mechanisms of azoxy bond remains unclear^4^.

**Fig. 1:**
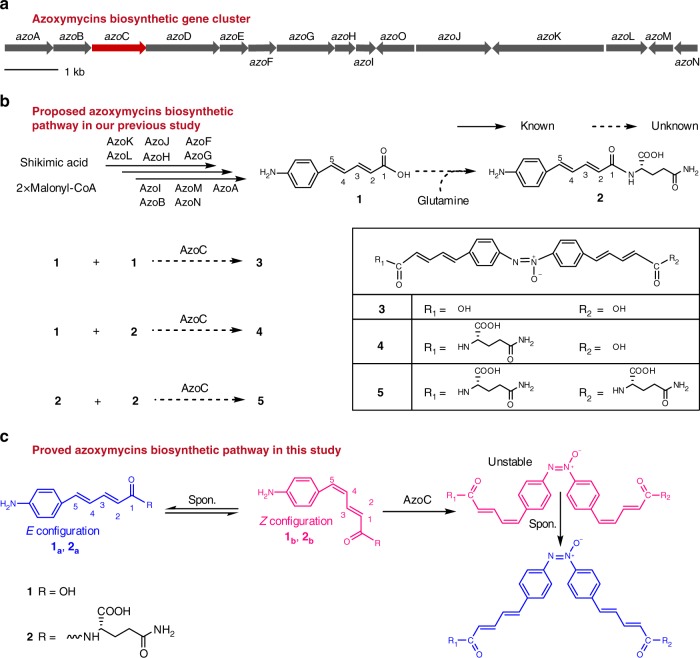
The biosynthesis of azoxymycins. **a** Organization of the azoxymycins biosynthetic gene cluster in *S. chattanoogensis*^5^. *azoC* is shown in red. **b** Proposed biosynthetic pathway of azoxymycins in our previous study^5^. AzoB, AzoF, AzoG AzoH, AzoI, AzoJ, AzoK, AzoL, AzoM, and AzoN are responsible for the formation of **1**, and AzoC is presumed to catalyze the azoxy bond formation. **c** The amended biosynthetic pathway of azoxymycins from this study

Previously, we had identified three aromatic azoxy natural products, azoxymycins A (**5**), B (**4**), and C (**3**), and their biosynthetic gene cluster from *Streptomyces chattanoogensis* (Fig. 1a, b)^5^. Interestingly, the deletion of *azoC*, a putative non-heme diiron *N*-oxygenase encoding gene within the cluster, caused the accumulation of two amine precursors (**1** and **2**), and the simultaneous disappearance of the azoxy compounds^5^. Thus, we speculate that AzoC is a key enzyme involved in azoxy bond formation.

Here, we show the biosynthetic mechanism for azoxy bond formation, which is initiated from the enzymatic oxidization of amine to the nitroso product catalyzed by non-heme diiron *N*-oxygenase AzoC. This is followed by the mutual conversion between nitroso and hydroxylamine groups, which is accelerated by coenzyme pairs. Eventually the azoxy bond is efficiently formed via dimerization of the nitrogen transient radicals, so long as both reducing and oxidizing coenzymes are provided. Our findings highlight that: (i) a non-heme diiron *N*-oxygenase AzoC authentically catalyzes the four-electron oxidation of amine to the nitroso group; (ii) redox coenzymes act as radical inducers in the activation of nitroso and hydroxylamine nitrogen atoms and increase their reaction activities; (iii) nitroso group is a key synthon in azoxy bond formation.

## Results

### Reconstruction of azoxy bond biosynthesis with AzoC

In silico analysis indicated that AzoC has two conserved iron-binding motifs (EX28-37DEXXH), and has 37 and 53% sequence identity to canonical non-heme diiron *N*-oxygenases AurF and CmlI (Supplementary Fig. 2), respectively, which form homodimers in solution and oxidize aromatic amine to nitro group during the biosynthesis of aureothin or chloramphenicol^6,7^. AzoC was expressed and purified from *Escherichia coli* BL21(DE3). Native-PAGE (non-denaturing polyacrylamide gel electrophoresis) and size-exclusion chromatography analysis showed that purified recombinant AzoC had formed homodimer in solution (Supplementary Fig. 3a, b). Moreover, the iron-to-enzyme ratio of AzoC was determined to be 1.78 using ferrozine assays (Supplementary Fig. 4), which was consistent with AurF and CmlI’s ratio of 2^6,7^. These data suggested that AzoC is also a non-heme diiron *N*-oxygenase.

**Fig. 2:**
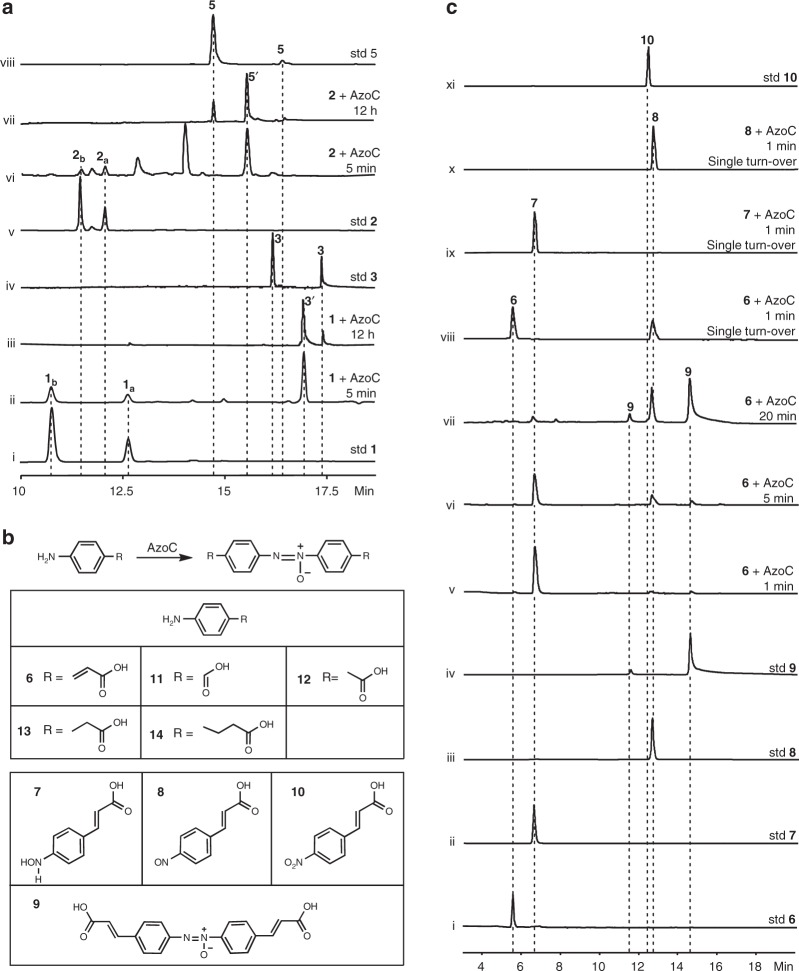
Catalytic activity of AzoC for azoxy bond formation. **a** HPLC analysis of in vitro azoxymycins biosynthesis catalyzed by AzoC at 370 nm. Reactions were performed in 20 mM HEPES buffer (pH 7) with the addition of 1 mM NADH and 10 μM PMS. (i) 100 μM **1**, (ii) 100 μM **1** with 10 μM AzoC for 5 min, (iii) 100 μM **1** with 10 μM AzoC for 12 h, (iv) standard azoxymycin C (**3**), the RT 16.2 min peak is the *trans* N = N isomer, and the RT 17.4 min peak is the *cis* N = N isomer, (v) 100 μM **2**, (vi) 100 μM **2** with 10 μM AzoC for 5 min, (vii) 100 μM **2** with 10 μM AzoC for 12 h, (viii) standard azoxymycin A, the RT 12.1 min peak is the *trans* N = N isomer, and the RT 13.8 min peak is the *cis* N = N isomer. **b** AzoC catalyzed azoxy bond formation, and related structures. **c** HPLC analysis of AzoC’s in vitro enzymatic reactions at 370 nm. Reactions were performed in 20 mM HEPES buffer (pH 7). (i) standard **6**, (ii) standard **7**, (iii) standard **8**, (iv) standard **9**, (v) 100 μM **6** with 1 μM AzoC for 1 min, (vi) 100 μM **6** with 1 μM AzoC for 5 min, (vii) 100 μM **6** with 1 μM AzoC for 20 min, (viii) single turn-over of **6** by incubation with 1 eq reduced AzoC for 1 min, (ix) single turn-over of **7** by incubation with 1 eq reduced AzoC for 1 min, (x) single turn-over of **8** by incubation with 1 eq reduced AzoC for 1 min, (xi) standard **10**

To investigate the catalytic activity of AzoC in azoxy bond biosynthesis, we purified precursors **1** and **2** from culture broth of *S. chattanoogensis ΔazoC*-mutant. High-performance liquid chromatography (HPLC) analysis showed that **1** and **2** were both mixtures of spontaneously inter-convertible C4 *E/Z* isomers with distinct ultraviolet/visible (UV/vis) spectra, which was in accordance with the reported literature about **1** and **2**^8^. Therefore, from here *E* isomers are designated as **1**_**a**_ and **2**_**a**_, and *Z* isomers are designated as **1**_**b**_ and **2**_**b**_ (Fig. 2a, Supplementary Figs. 5 and 6). We then performed in vitro biochemical assays by incubating 10 μM purified AzoC with 100 μM **1**, 1 mM NADH, and 10 μM phenazine methosulfate (PMS, a chemical reductant used to restore the activity of diiron *N*-oxygenase) in 20 mM (pH 7) HEPES (4-(2-hydroxyethyl)-1-piperazineethanesulfonic acid) buffer^9^. HPLC analysis of our time-course experiments showed that **1** was predominantly transformed into a major product with an identical molecular weight, but a different retention time, to **3** in 5 min (peak **3′** in Fig. 2a, ii, and Supplementary Fig. 7). However, **3** was not detected until 12 h later (Fig. 2a, iii). Also note that symmetric azoxy compound shows two peaks with different UV/vis spectra according to HPLC, due to the spontaneous mutual *E/Z* isomerization of the N = N bond^1,5^). Given that **1** had mutual C4 *E/Z* isomerization and that **3′** had the same molecular formula as **3** according to high-resolution mass spectrometry analysis, we proposed that **3′** might be the corresponding C4 *E/Z* isomer of **3**. This hypothesis was supported by our time-course experiments and dynamic study, both of which indicated that AzoC consumed **1**_**b**_ faster than **1**_**a**_, and caused the resulting azoxy product to be the corresponding C4 isomers (**3′** and **5′**, Supplementary Fig. 9). The same result was also observed in the in vitro experiments of **2** (Fig. 2a, v–viii, Supplementary Figs. 8–9). Based on these data, the biosynthetic pathway of azoxymycins was amended (Fig. 1c). Meanwhile, we also found that AzoC could convert other *p*-aminobenzene compounds (**6**, **11**, **12**, **13**, **14**) to their corresponding azoxy analogs (Fig. 2b and Supplementary Figs. 10–14). These results suggest that AzoC has the catalytic activity for the biosynthesis of azoxy bond from amine substrates.

### AzoC authentically oxidizes amine to nitroso group

To explore the detailed biosynthetic pathway of azoxy bond in azoxymycins, we chose **6** as a model substrate as it is structurally similar to **1** but has no *E/Z* isomerization. The hydroxylamine (**7**), nitroso (**8**), and azoxy (**9**) analogs of **6** were chemically synthesized (Supplementary Figs. 15–20 and Supplementary Table 2)^10^, while the nitro analog (**10**) was commercially available (Fig. 2c, xi). With **6** as model substrate, the steady-state kinetic parameter of AzoC was determined, and *K*_cat_ = 42.03 ± 4.19 min^−1^, *K*_m_ = 4.24 ± 0.9 μM, and *K*_cat_/*K*_m_ = 10.15 ± 1.16 min^−1^ μm^−1^ at 30 °C. To fully elucidate the reaction process, both time-course (Fig. 2c, v–vii) and single turn-over (Fig. 2c, viii–x) in vitro experiments were performed^11^.

In attempt to slow down the reaction rate and capture the possible reaction intermediates, the concentration of AzoC was decreased to 1 μM in the time-course experiments. We found that **6** was rapidly converted to **7** within 1 min (Fig. 2c, v). After 5 min, **8** and a small amount of **9** were produced (Fig. 2c, vi). With further incubation to 20 min, primary accumulation of **8** and **9** with a remaining trace amount of **7** was detected (Fig. 2c, vii). In all these reactions, no production of **10** was observed (Fig. 2c). These results seemed to suggest that **7** was the first oxidative intermediate, and the nitroso compound **8** was the second intermediate in azoxy bond biosynthesis. However, this catalytic route is still questionable as there was no nitroso or hydroxylamine analog of **1** and **2** detected in *S. chattanoogensis*.

Single turn-over experiments were then performed. According to the literature, reduced diferrous diiron *N*-oxygenase shows featureless optical spectrum, but when exposed to oxygen the reduced enzyme quickly turned to peroxo state and show new optical absorbance at ~500 nm^11^. With this characteristic, we analyzed the absorbance spectrum of reduced AzoC (reduced by PMS and ascorbic acid, and excessive reducing reagents were removed by washing three times with an equal volume of deoxygenated HEPES buffer on a PD-10 column). The result showed that a clear absorbance band at around 500 nm had emerged after reduced AzoC exposed to oxygen (Supplementary Fig. 21). This result also proved the reducing and peroxo state of AzoC and indicated that the reduced enzyme was suitable for the subsequent single turn-over experiments. In the single turn-over experiments, fully reduced AzoC was incubated with equivalent of **6**, **7**, or **8** in oxygen-saturated HEPES buffer, respectively, for 1 min (without NADH). HPLC analysis results indicated that **6** could only be transformed to **8** (Fig. 2c, viii) but not to **7**. Meanwhile, neither **7** nor **8** could be further oxidized by AzoC (Fig. 2c, ix–x). As the single turn-over reaction only contained enzyme and substrate, and thus enable the exclusion of any other reagents’ impact, these results provide stronger evidence in the catalytic activity study of AzoC.

These experiments suggested that the direct four-electron oxidization of amine to nitroso group was the authentic activity of AzoC, and that this enzyme could not catalyze the oxidization of hydroxylamine or the nitroso group. However, questions still remain to explain the accumulation of **7** in the time-course experiments and the transformation of **8** to **9**.

### Non-enzymatic azoxy bond formation mediated by coenzymes

**Fig. 3:**
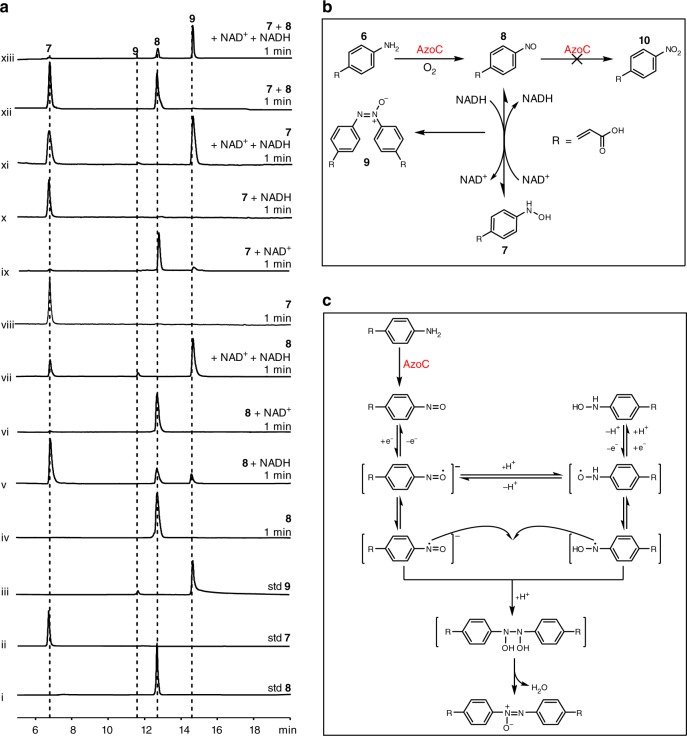
Biosynthetic pathway of azoxy bond. **a** HPLC analysis of in vitro non-enzymatic reactions at 370 nm. Reactions were performed in 20 mM HEPES buffer (pH 7). (i) Standard **8**; (ii) standard **7**; (iii) standard **9**; (iv) **8** in buffer for 1 min; (v) **8** with 10 eq NADH in buffer for 1 min; (vi) **8** with 10 eq NAD^+^ in buffer for 1 min; (vii) **8** with 10 eq NAD^+^ and 10 eq NADH in buffer for 1 min; (viii) **7** in buffer for 1 min; (ix) **7** with 10 eq NAD^+^ in buffer for 1 min; (x) **7** with 10 eq NADH in buffer for 1 min; (xi) **7** with 10 eq NAD^+^ and 10 eq NADH in buffer for 1 min; (xii) **7** + **8** in buffer for 1 min; (xiii) **7** + **8** with 10 eq NAD^+^ and 10 eq NADH in buffer for 1 min. **b** Biosynthetic scheme of **9** from **6**. AzoC enzymatically oxidizes **6** to **8**, and then NAD^+^/NADH induces non-enzymatic reciprocal transformation between **7** and **8**. **9** is then formed during the redox reaction process. **c** Proposed radical biosynthetic mechanism of azoxy bond from nitroso group

These questions left in the time-course and single turn-over experiments prompted us to assume that the accumulation of **7** might be caused by a yet unknown non-enzymatic reaction independent of AzoC. By comparing two reaction systems, we found that there was excess NADH (10 equimolar (eq)) in the time-course reaction system, which might be responsible for the transformation of **8** to **7**. Thus, we attempted to test whether NADH could cause the transformation from **8** to **7** or **9**. With HPLC analysis, we found most of **8** was transformed to **7** when incubated only with 10 eq NADH for 1 min, along with slight production of **9** (Fig. 3a, v). Meanwhile, no transformation was observed when **8** was incubated with 10 eq NAD^+^ (Fig. 3a, vi). Consistently, **7** could be transformed to **8** and a small amount of **9** when incubated only with 10 eq NAD^+^ for 1 min (Fig. 3a, ix), and NADH had no influence on **7** (Fig. 3a, x). We further analyzed NAD^+^ and NADH changing dynamics by measuring the characteristic absorbance of NADH at 340 nm. Results showed that the NADH level had reduced by ~15% within 1 min when incubated with **8**, and that ~17% of NAD^+^ was transformed to NADH when it was incubated with **7** (Supplementary Fig. 22). These data together suggest that NAD^+^ and NADH are the true redox reagents that non-enzymatically facilitate the transformation between hydroxylamine and nitroso groups.

The trace of **9** that was formed during the transformation between **7** and **8** also requires explanation. It had been proposed that aromatic azoxy bond might be formed by condensation of nitroso and hydroxylamine building blocks^12^. We initially hypothesized that **9** could be dimerized from **7** and **8**. However, due to the fact that no **9** was detected when **7** + **8** were incubated without NAD^+^ or NADH for 1 min (Fig. 3a, xii), we excluded this assumption. We then hypothesized that azoxy bond might be formed from the transient intermediates of niroso and hydroxylamine products during their mutual conversion process induced by coenzymes. Therefore, we designed the experiment by incubating **7** or **8** individually, or **7** + **8** with both 10 eq NAD^+^ and 10 eq NADH. Surprisingly we found that **9** was predominantly produced with the consumption of most of the substrates within 1 min (Fig. 3a, xi, vii, and xiii).

All the above reactions were performed with model compounds **7**, **8**, and **9**. We next synthesized three other aromatic nitroso compounds (*p*-nitroso benzoic acid, *p*-nitroso phenylacetic acid, and *p*-nitroso phenypropionic acid, Supplementary Figs. 23–25) and tested them with NAD^+^/NADH. HPLC analysis showed that these nitroso compounds could also be efficiently converted to their azoxy dimers within 1 min when incubating with both NAD^+^ and NADH (Supplementary Figs. 26–28). With these experiments, the biosynthetic scheme from amine to azoxy product is strongly concluded (Fig. 3b).

The conversion of hydroxylamine to the nitroso group is a two-electron oxidization process. Theoretically the two-electron oxidization can be divided to 1 electron + 1 electron processes, during which free radicals might be formed to facilitate the dimerization for the azoxy bond^13,14^. To verify this hypothesis, we introduced 2,2,6,6-tetramethyl-1-piperidinyloxy and butylated hydroxytoluene (TEMPO and BHT, two typical radical scavengers), respectively, into the reaction, and found that both TEMPO and BHT could inhibit the conversion of **8** or **7** to **9** (Supplementary Fig. 29). Based on this, a radical-mediated biosynthetic mechanism from nitroso group to azoxy bond is proposed (Fig. 3c).

NAD^+^/NADH is only one of the redox coenzyme pairs in the cellular metabolism^15^. Therefore, we sought to check whether other redox coenzyme pairs could mediate hydroxylamine and nitroso group transformation for subsequent azoxy bond formation by testing **7** or **8** with other coenzymes (NADP^+^/NADPH, FAD/FADH_2_, FMN/FMNH_2_). We found that **7** could be efficiently oxidized to **8** when only incubated with NADP^+^, FAD, or FMN, while **8** could be readily reduced to **7** when only incubated with NADPH, FADH_2_, or FMNH_2_, all of which was accompanied by the production of **9** (Supplementary Fig. 30, i–iii, and v–vii). Furthermore, if both reductive and oxidative coenzymes (NADP^+^/NADPH, FAD/FADH_2_, or FMN/FMNH_2_) were provided, **7** or **8** alone could be efficiently and predominantly transformed into **9** (Supplementary Fig. 30, ix–xi and xiii–xv). Interestingly, we found that the non-coenzyme redox pair, H_2_O_2_/sodium dithionite, also had a similar role in the production of **9** (Supplementary Fig. 30 iv, viii, xii, and xvi). These phenomena further supported our non-enzymatic radical reaction model for azoxy bond formation.

### Mechanism study of AzoC’s catalytic activity

**Fig. 4:**
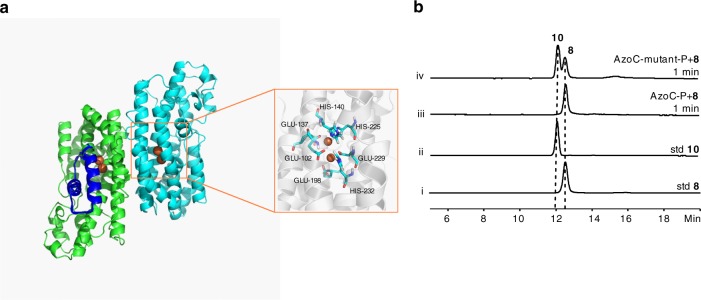
In silico model of AzoC and reconstitution of nucleophilic activity of AzoC via sequence shuffling. **a** Structure model of AzoC. The iron atoms are shown as brown spheres. The two iron ions are coordinated with protein residues Glu-102, Glu-137, His-140, Glu-198, His- 225, Glu-229, and His-232.The mutated sequence of AzoC-mutant is shown as a blue ribbon. **b** HPLC analysis of AzoC-P and AzoC-mutant-P’s in vitro biochemical reactions at 345 nm. Reactions were performed in 20 mM HEPES buffer (pH 7). (i) Standard **8**, (ii) standard **10**, (iii) **8** with eq AzoC-P for 1 min, (iv) **8** with eq AzoC-mutant-P for 1 min

Canonical non-heme diiron *N*-oxygenases (AurF and CmlI) have been reported to catalyze the successive 2 electron + 2 electron + 2 electron oxidation of amine to the nitro group^16,17^. In challenging this canonical oxidation model, a new model based on theoretical calculation was recently reported, which could better explain the reaction details of AurF and CmlI^18^. It was proposed that the oxygenated intermediate centers (P) of AurF and CmlI are ambiphilic hydroperoxo species, and that P acts as electrophilic reagent to grab the hydrogen atom from amine and cause the direct oxidization of amine to the nitroso group in the first step (without the formation of hydroxylamine). Subsequently, proton-coupled electron transfer from hydroperoxo P generates a superoxo species, which acts as nucleophilic reagent to attack the nitroso nitrogen atom for the formation of nitro^18^. The direct four-electron oxidation of amine to nitroso group in our study corresponds to the theoretical predication of non-heme diiron *N*-oxygenase^18^. While AzoC showed a 37% sequence identity to AurF, we presumed that AzoC-P was not as nucleophilic as AurF-P in proceeding the oxidization of the nitroso group to nitro. We then expressed and purified AurF, and tested its oxidization of nitroso product **8**. Our single turn-over results showed that AurF-P could oxidize **8** to **10**, while AzoC-P did not have this catalytic ability (Supplementary Fig. 31b). In the theoritical calculation modeling^18^, the nucleophilic attack of P on the nitroso group was coupled with an electron transfer to E196 (glutamic acid 196) bonding Fe. The relevant Fe was bonded to the E198-H232 sequence of AzoC (Supplementary Fig. 31a). We then wondered if the nucleophilic activity could be restored by sequence shuffling. So we constructed an AzoC-mutant by replacing its E198-H232 sequence to the corresponding sequence of AurF. The resulting AzoC-mutant-P was subject to in vitro reactions with compound **8**. As shown in Fig. 4b, the mutant was able to catalyze the oxidization of **8** to **10**, indicating that the nucleophilic reactivity was partly restored. Next, we constructed an in silico structure model of AzoC (Fig. 4a and Supplementary Fig. 31), and used the model and crystal structure of AurF (PDB code 3chh.A) for docking experiments (compound **8** was used as a docking substrate, Supplementary Fig. 31c–e). The modeling results showed that the relative distance and location of AzoC’s E198 to Fe and **8** (Supplementary Fig. 31d) was significantly different to that of AurF (Supplementary Fig. 31c). However, that of AzoC-mutant was more similar to the model of AurF. The in vitro biochemical data with shuffling proved that the oxidative activity of non-heme diiron oxygenase, especially the oxidative activity of the nitroso group to nitro by AzoC, could be restored. The modeling results imply that the E198’s binding model and relative location within the active center might be the mechanism behind AzoC’s nucleophilic deficiency in oxidizing the nitroso group to nitro.

## Discussion

Here, we have reported a unique azoxy bond biosynthetic mechanism composed of an enzymatic nitroso moiety-producing reaction and a non-enzymatic N–N bond coupling reaction. The enzyme reaction mechanism was proposed using in silico modeling and docking experiments, while the non-enzymatic radical coupling azoxy bond formation mechanism was confirmed using the radical scavenger inhibition experiments.

AzoC, which catalyzes the oxidization of amine to the nitroso group, is identified as a non-heme diiron *N*-oxygenase. To date, there has been only a few non-heme diiron *N*-oxygenases reported, most of which catalyze the six-electron oxidization of amine to nitro^19^. Recently, it has been reported that non-heme diiron *N*-oxygenase TsnB7^20^ and BezJ^21^ are capable of the oxidization of glutamine and *p*-amino benzoic acid’s amines to their hydroxylamine analogs. In addition, AurF was also seen to catalyze the oxidization of *p*-hydroxylamine benzoic acid to *p*-nitro benzoic acid^22^. These results reveal the diversity of non-heme diiron *N*-oxygenase’s catalytic tool kit in nitrogen-containing bond biosynthesis. However, the nitroso-producing activity of non-heme diiron *N*-oxygenase has not yet been reported. In HEDM and other nitrogen-containing heterocycle synthesis, nitroso groups have better chemical activities than nitro, and they can act as both nucleophilic and electrophilic reagent to easily install nitrogen and oxygen atom complex structures^23–26^, such as imidaze^27^, benzimidazole^28^, triazole^29^, tetrazine^30^, and azabicycle^31^, all of which are synthetic intermediates in HEDM. Moreover, nitroso groups are good starting synthons in the synthesis of diazo, azo, and azoxy bond, which are efficient enhancers to significantly increase the energy level and thermal stability of HEDM^1^. In this way, our discovery of a nitroso production catalyzed by AzoC represents an efficient enrichment of non-heme diiron *N*-oxygenase’s catalytic portfolio and sheds light onto the biosynthesis of complex nitrogen-containing heterocycles, especially in HEDM biosynthesis.

In organic synthesis, N–N bond synthesis is performed at harsh reaction conditions with low efficiency, and with uncontrollable regio- and sterio-selectivity^32–34^. Hence, biosynthesis is an promising alternative method for the generation of N–N bonds. There have already been several aspects of N–N bond biosynthesis reported, such as the diazo bond biosynthetic pathway in cremeomycin biosynthesis^35^, the hydrazine N–N bond biosynthesis of fosfazinomycin and kanamycin^36^, the N–N bond biosynthesis of piperazate^37^; the hydrazine biosynthesis machinery of s56-p1^38^; and the non-enzymatic N–N bond formation of dixiamycin A and B mediated by a flavoenzyme^39^. In the biosynthesis of these N–N bond, either nitrous acid^35,36^ or a special enzyme^37–39^ are employed to activate the nitrogen atom to form N–N bond. Nitrous acid is easy to diffuse in water and very vulnerable to be neutralized in living cells, so it is very hard to control in biosynthesis. The enzyme activation of the nitrogen atom significantly relies on the substrate specificity of the enzyme. The compatibility with other biosynthetic units is also a critical issue in biosynthetic route design. While the activation of the nitrogen atom is the key point in N–N bond biosynthesis, a controllable and biosynthetically benign nitrogen-activating method would be more acceptable to synthetic biologists. As generally accepted assistant molecules, coenzymes are both biocompatible and controllable with various bioengineering methods^40–42^. Hence, the activation of nitroso nitrogen atom by coenzymes in our azoxy bond biosynthesis provides the possibility to synthesize N–N bond in aqueous biosystem and in mild conditions. This represents a considerable step forwards in our understanding about azoxy N–N bond biosynthesis, and sheds light on the biosynthesis of valuable azoxy compounds, such as HEDM.

In conclusion, we have elucidated that azoxy bond is biosynthesized via an enzymatic and non-enzymatic coupling cascade reaction. Our mechanism discovery illuminates the biosynthetic mechanism of azoxy bond, and makes the biosynthesis of azoxy compounds possible. Thus, it could provide a technical basis for the scale-up industrial biosynthesis of HEDM and other precious azoxy compounds.

## Methods

### General methods

Primers were purchased from Shanghai Generay Biotech. Reagents were purchased from Sigma-Aldrich, J&K Chemical, and Aladdin Bio-Chem Technology.

### Gene cloning

*azoC* gene was amplified by PCR from genomic DNA of *S. chattanoogensis* with primers P1 and P2. Then, the fragment was cloned into *Nde*I*/Hin*dIII-digested pET28a vector to obtain the expression plasmid pET28a-*azoC*. pET28a-*aurF* was synthesized by Generay (Shanghai, China). The *azoC*-mutant was amplified by PCR from the plasmid pET28a-*azoC* with primers P3–P4 for fragment 1 and P7–P8 for fragment 3 and from the plasmid pET28a-*aurF* with primers P5–P6 for mutant fragment 2. Then, the three fragments were cloned into pET28a vector using a CloneExpress II Multis Cloning Kit (Vazyme, Nanjing, China) to obtain the expression plasmid pET28a-*azoC*-mutant.

Primer list:

P1: 5′-CATATGATGAGCAGTCGGGCGCCCG-3′;

P2: 5′-AAGCTTTCAGCGAGTGGGGGAGACGG-3′;

P3: 5′-ctggtgccgcgcggcagcCATATGATGAGCAGTCGGGCGCCC-3′;

P4: 5′-ggcgttgatgcaggtctcGGCCACGGTGGCGAAGGC-3′;

P5: 5′-GAGACCTGCATCAACGCCCT-3′;

P6: 5′-caccgcggagatcgaggcGTGCGCCGTCTCGTCCCG-3′;

P7: 5′-GCCTCGATCTCCGCGGTGC-3′;

P8: 5′-gtgctcgagtgcggccgcAAGCTTTCAGCGAGTGGGGGAGACGG-3′.

(Note: the underlined sequences in primers refer to the restriction enzyme cutting site, and lower case sequences meant the homologous sequence for CloneExpress II Multis cloning.)

### Protein expression and purification

*Escherichia coli* BL21(DE3) was transformed by the plasmid harboring each gene and grown in 1 L of lysogeny broth (LB) supplemented with 1 mM ammonium ferric sulfate at 37 °C with shaking at 220 r.p.m. until an OD_600_ of 0.6 was reached. The culture was added with 0.1 mM isopropyl β-d-1-thiogalactopyranoside for induction overnight at 25 °C. Cells were collected and disrupted in buffer A (20 mM Tris, pH 8.0, 250 mM NaCl, and 10 mM imidazole) by sonication. Soluble protein was purified on Ni^2+^-nitrilotriacetic acid resin and eluted with buffer A solution containing 250 mM imidazole. After that, recombinant protein was desalted on a 10-kDa YM-3 column with 20 mM Tris buffer (pH 7.5). Protein concentration was determined with the Bradford assay and bovine serum albumin as a standard.

### Determination iron-to-enzyme ratio of AzoC (ferrozine assay)

Fifty microliters of protein solution (20–200 μM range) was mixed with an equal volume of reducing reagent and incubated at room temperature for 5 min, followed by the addition of 50 μL of 12% trichloroacetic acid. After centrifugation, 100 μL of supernatant was transferred to 40 μL of 10% ammonium acetate buffer, followed by the addition of 10 μL of ferroin reagent. The mixture was incubated for 10 min at room temperature, and the absorbance was subsequently measured at 562 nm. Ammonium ferrous sulfate was used to prepare the standard solutions, and molar extinction coefficient constant at 562 nm was 28,000 M^−1^ cm^−1^. The reducing reagent consists of 48 μL of A [ascorbic acid (4 mM), H_2_SO_4_ (0.1 M)] and 2 μL of B [PMS (10 mM)]. The ferroin reagent is composed of ferrozine (6 mM) and H_2_SO_4_ (0.05 M).

### Size-exclusion chromatography analysis

The molecular weight of AzoC was determined by an AKTA Purifier system equipped with Sephacryl^TM^S-300 column. The parameters were: 4 °C; solvent, 20 mM Tris, pH 8.0, 250 mM NaCl; flow rate, 1 mL min^−1^; UV absorbance at 215 nm. Protein markers used were IgG (150 kDa) and bovine serum albumin (67 kDa).

### AzoC in vitro reaction system

In vitro biochemical reactions were performed by incubating the purified enzyme with 100 μM substrates, 1 mM NADH, and 10 μM PMS in 20 mM HEPES buffer (pH 7) at 30 °C.

### Single turn-over experiment

AzoC was reduced with eq PMS and 4 eq ascorbic acid anaerobically. Then, PMS and ascorbic acid were removed using a PD‐10 desalting column (G‐25, GE Healthcare). After that, reduced AzoC was mixed with eq substrate(s) in O_2_-saturated 20 mM HEPES buffer (pH 7) under anaerobic condition, and further incubated at 30 °C for 1 min.

### Generation of AzoC-P and AurF-P

AzoC (2 mM) or AurF (2 mM) was reduced under anaerobic conditions with an excess amount of ascorbic acid in the presence of 0.1 equivalent of PMS. Excess reductant and PMS were removed using a PD‐10 desalting column (G‐25, GE Healthcare) in an anaerobic chamber. Reduced AzoC or AurF were exposed to O_2_ at ~4 °C for 1 min, and used immediately for subsequent reactions.

### HPLC analysis method

HPLC analysis of in vitro reactions was carried out on Agilent 1260 infinity system with a DAD detector at 370 nm. Column used was Agilent ZORBAX SB-C18 (5 μm, 4.6 × 150 mm^2^). Mobile phase A was 0.1% triflouroacetic acid (TFA) in water, and mobile phase B was 0.1% TFA in acetonitrile. Flow rate was 1 mL min^−1^. During the analysis procedure, mobile phase B was raised from 10 to 75% in 20 min.

### Fast HPLC analysis method

Analysis was carried out on Agilent 1260 infinity system with a DAD detector at 370 nm. Column used was Agilent Poroshell SB-C18 (2.7 μm, 4.6 × 7.5 mm^2^). Mobile phase A was 0.1% TFA in water, and mobile phase B was 0.1% TFA in acetonitrile. Flow rate was 0.5 mL min^−1^. During the analysis procedure, mobile phase B was raised from 10 to 75% in 5 min.

### Synthesis of **7**

A mixture of 0.25 g of **10** and a solution of 50 mL of 3% ammonium chloride was stirred vigorously on ice, and 0.3 g of zinc dust was added in small portions over a period of 5 min. Twenty minutes later, the solution was filtered and frozen dried (all processes should be carried out at low temperature and avoid light). After that, the residue was subjected to preparative HPLC.

### Synthesis of **8**

One milliliters of pre-cold sulfuric acid solution was added to 25 mL of **7**’s pre-cold solution (~2 g L^−1^). Then, 0.1 g of sodium dichromate dihydrate was added (all processes were carried out on ice). After 2 min, the solution was neutralized and subjected to Sephadex LH-20 gel.

### Synthesis of **9**

A measure of 0.86 g of potassium borohydride, 0.4 g of PEG-400, and 0.2 g of **10** were added to 20 mL of 2% KOH aqueous solution. The reaction was taken at 80 °C for 12 h in a stirring flask equipped with a condenser. After that, the extract was neutralized and referred to Sephadex LH-20 gel and preparative HPLC.

### Synthesis of *p*-nitroso phenyl acids

A mixture of 0.25 g of *p*-nitro benzoic acid (or *p*-nitro phenylacetic acid or *p*-nitro phenypropionic acid) and a solution of 50 mL of 3% ammonium chloride was stirred vigorously on ice. A measure of 0.3 g of zinc dust was added over a period of 5 min. Twenty minutes later, the solution was filtered. Then, 2 mL of pre-cold sulfuric acid solution was added to the solution, and 0.2 g of sodium dichromate dihydrate was added subsequently (all processes were carried out on ice). After 2 min, the solution was neutralized and subjected to Sephadex LH-20 gel.

### Molecular modeling and docking analysis

The protein structure of AzoC was constructed with Modeller 9.18. The crystallographic structure of AurF (Protein Data Bank (PDB) code 3chh.1A) was chosen as tertiary structural template showing 37% sequence identity. The resulting model was refined by energy minimization using Gromacs 5.1.4. AzoC-mutant was constructed by replacing its E198-H232 sequence to the corresponding sequence of AurF, and the structure model was constructed with Modeller 9.18. The generated models (AzoC and AzoC-mutant) and the crystal structure of AurF (PDB, code 3chh.A) were used for docking of compound **8**, respectively. Ligand (compound **8**) was energy optimized using MM2 energy minimization (ChemBio3D, CambridgeSoft). Graphical User Interface program AutoDock Tools was used to prepare the ligand and receptor. AutoGrid was used for the preparation of the grid map using a grid box. For AzoC, grid box parameters (*x*, *y*, *z*): (42.932, 43.085, 42.225); size (48, 34, 26). For AurF, grid box parameters (*x*, *y*, *z*): center (−5.235, 0.189, −2.74); size (44, 34, 26). For AzoC-mutant, grid box parameters (*x*, *y*, *z*): center (42.88, 42.691, 43.734); size (46, 32, 26). AutoDock/Vina was employed for docking using protein and ligands information along with grid box properties in the configuration file. During the docking procedure, both the protein and ligands are considered as rigid. The results <1.0 Å in positional root-mean-square deviation were clustered together and represented by the result with the most favorable free energy of binding. The pose with lowest energy of binding or binding affinity was extracted and aligned with receptor structure. The obtained docked poses were analyzed using PyMOL.

### Reporting summary

Further information on research design is available in the Nature Research Reporting Summary linked to this article.

## Supplementary information

Supplementary Information

Reporting Summary

Source Data

## Data Availability

The data underlying the findings of this study are available in this article, Supplementary Information files, and from the corresponding authors upon request. Source data underlying Supplementary Figs. 3, 4a, b are available as a separate file. The sequences of AzoC have been deposited to GenBank (AKQ24642.1). Correspondence and requests for materials should be addressed to Y.Q.L and X.M.M. (email:lyq@zju.edu.cn and xmmao@zju.edu.cn)

## References

[1] Dai YT (2018). Light-tuned selective photosynthesis of azo- and azoxy-aromatics using graphitic C_3_N_4_. Nat. Commun..

[2] Yin P, Zhang QH, Shreeve JM (2016). Dancing with energetic nitrogen atoms: versatile N-functionalization strategies for *N*-heterocyclic frameworks in high energy density materials. Acc. Chem. Res..

[3] Wang Y (2018). Accelerating the discovery of insensitive high-energy-density materials by a materials genome approach. Nat. Commun..

[4] Waldman AJ, Ng TL, Wang P, Balskus EP (2017). Heteroatom–heteroatom bond formation in natural product biosynthesis. Chem. Rev..

[5] Guo YY (2015). Identification and biosynthetic characterization of natural aromatic azoxy products from *Streptomyces chattanoogensis* L10. Org. Lett..

[6] Lu HG, Chanco E, Zhao HM (2012). CmlI is an *N*-oxygenase in the biosynthesis of chloramphenicol. Tetrahedron.

[7] Krebs C, Matthews ML, Jiang W, Bollinger JM (2007). AurF from *Streptomyces thioluteus* and a possible new family of manganese/iron oxygenases. Biochemistry.

[8] Potterat O, Zahner H, Metzger JW, Freund S (1994). Metabolic products of microorganisms. Part 269. 5-Phenylpentadienoic-acid derivatives from *Streptomyces* sp. Helv. Chim. Acta.

[9] Choi YS, Zhang H, Brunzelle JS, Nair SK, Zhao H (2008). In vitro reconstitution and crystal structure of *p*-aminobenzoate *N*-oxygenase (AurF) involved in aureothin biosynthesis. Proc. Natl. Acad. Sci. USA.

[10] Liu YF (2011). Reduction of nitroarenes to azoxybenzenes by potassium borohydride in water. Molecules.

[11] Makris TM (2015). An unusual peroxo intermediate of the arylamine oxygenase of the chloramphenicol biosynthetic pathway. J. Am. Chem. Soc..

[12] Ding L (2012). Elaiomycins D–F, antimicrobial and cytotoxic azoxides from *Streptomyces* sp. strain HKI0708. J. Nat. Prod..

[13] Zuman P, Shah B (1994). Addition, reduction, and oxidation reactions of nitrosobenzene. Chem. Rev..

[14] Russell GA, Geels EJ (1965). Paramagnetic intermediates in the condensation of nitrosobenzene and phenylhydroxylamine. J. Am. Chem. Soc..

[15] Walsh CT, Tu BP, Tang Y (2017). Eight kinetically stable but thermodynamically activated molecules that power cell metabolism. Chem. Rev..

[16] Jayapal P, Ansari A, Rajaraman G (2015). Computational examination on the active site structure of a (peroxo)diiron(III) intermediate in the amine oxygenase AurF. Inorg. Chem..

[17] Jasniewski AJ, Komor AJ, Lipscomb JD, Que L (2017). Unprecedented (*μ*-1,1-peroxo)diferric structure for the ambiphilic orange peroxo intermediate of the nonheme *N*-oxygenase Cmll. J. Am. Chem. Soc..

[18] Wang C, Chen H (2017). Convergent theoretical prediction of reactive oxidant structures in diiron arylamine oxygenases AurF and CmlI: peroxo or hydroperoxo?. J. Am. Chem. Soc..

[19] Komor AJ, Jasniewski AJ, Que L, Lipscomb JD (2018). Diiron monooxygenases in natural product biosynthesis. Nat. Prod. Rep..

[20] Kudo K (2017). Biosynthetic origin of the hydroxamic acid moiety of trichostatin a: identification of unprecedented enzymatic machinery involved in hydroxylamine transfer. J. Am. Chem. Soc..

[21] Tsutsumi H (2018). Unprecedented cyclization catalyzed by a cytochrome P450 in benzastatin biosynthesis. J. Am. Chem. Soc..

[22] Li N (2010). Four-electron oxidation of *p*-hydroxylaminobenzoate to *p*-nitrobenzoate by a peroxodiferric complex in AurF from *Streptomyces thioluteus*. Proc. Natl Acad. Sci. USA.

[23] Shaum JB, Fisher DJ, Sroda MM, Limon L, Read de Alaniz J (2018). Direct introduction of nitrogen and oxygen functionality with spatial control using copper catalysis. Chem. Sci..

[24] Wang PC, Xu YG, Lin QH, Lu M (2018). Recent advances in the syntheses and properties of polynitrogen pentazolate anion *cyclo*-N_5_^−^ and its derivatives. Chem. Soc. Rev..

[25] Millar RW, Philbin SP, Claridge RP, Hamid J (2004). Studies of novel heterocyclic insensitive high explosive compounds: pyridines, pyrimidines, pyrazines and their bicyclic analogues. Propellants Explos. Pyrotech..

[26] Li YM, Chakrabarty S, Studer A (2015). An efficient approach to chiral allyloxyamines by stereospecific allylation of nitrosoarenes with chiral allylboronates. Angew. Chem. Int. Ed..

[27] He S, Tan GY, Luo AP, You JS (2018). Rhodium-catalyzed oxidative C–H/C–H cross-coupling of aniline with heteroarene: *N*-nitroso group enabled mild conditions. Chem. Commun..

[28] Manna S, Matcha K, Antonchick AP (2014). Metal-free annulation of arenes with 2-aminopyridine derivatives: the methyl group as a traceless non-chelating directing group. Angew. Chem. Int. Ed..

[29] Gallos JK, Lioumi MI, Lekka AN (1993). The reaction of 2-nitrosopyridine with nitrile oxides: first synthesis of 1,2,4-triazolo[1,5-*a*]pyridine 1,3-Di-N-oxides. J. Heterocycl. Chem..

[30] Powell BF, Overberger CG, Anselme J-P (1983). Hydrosulfite reduction of *N*-nitroso-1,2,3,4-etrahydroisoquinolines and oxidation of *N*-amino-l,2,3,4 tetrahydroisoquinolines. J. Heterocycl. Chem..

[31] Maji B, Yamamoto H (2015). Catalytic enantioselective nitroso Diels–Alder reaction. J. Am. Chem. Soc..

[32] Liu YG (2016). Bis(4-nitraminofurazanyl-3-azoxy)azofurazan and derivatives: 1,2,5-oxadiazole structures and high-performance energetic materials. Angew. Chem. Int. Ed..

[33] Yin P, Parrish DA, Shreeve JnM (2014). *N*-diazo-bridged nitroazoles: catenated nitrogen-atom chains compatible with nitro functionalities. Chemistry.

[34] Taylor KG, Isaac SR, Clark MS (1976). Aliphatic azoxy compounds. IV. Reaction of nitrosoalkanes with hydroxylamines. Synthesis of unsymmetrical primary and secondary azoxyalkanes by nitrogen–nitrogen bond formation. J. Org. Chem..

[35] Sugai Y, Katsuyama Y, Ohnishi Y (2016). A nitrous acid biosynthetic pathway for diazo group formation in bacteria. Nat. Chem. Biol..

[36] Wang K-KA (2018). Glutamic acid is a carrier for hydrazine during the biosyntheses of fosfazinomycin and kinamycin. Nat. Commun..

[37] Du YL, He HY, Higgins MA, Ryan KS (2017). A heme-dependent enzyme forms the nitrogen-nitrogen bond in piperazate. Nat. Chem. Biol..

[38] Matsuda K (2018). Discovery of unprecedented hydrazine-forming machinery in bacteria. J. Am. Chem. Soc..

[39] Zhang Q (2017). Characterization of the flavoenzyme XiaK as an *N*-hydroxylase and implications in indolosesquiterpene diversification. Chem. Sci..

[40] Cracan V, Titov DV, Shen HY, Grabarek Z, Mootha VK (2017). A genetically encoded tool for manipulation of NADP^+^/NADPH in living cells. Nat. Chem. Biol..

[41] Ling C (2018). Engineering NADH/NAD^+^ ratio in *Halomonas bluephagenesis* for enhanced production of polyhydroxyalkanoates (PHA). Metab. Eng..

[42] Verdin E (2015). NAD^+^ in aging, metabolism, and neurodegeneration. Science.

