# Supplementary material for: Molecular mechanism of azoxy bond formation for azoxymycins biosynthesis
Source: Nat Commun. 2019 Oct 8;10:4420. doi: 10.1038/s41467-019-12250-1 (PMC6783550; doi:10.1038/s41467-019-12250-1)
Supplement: Supplementary file 1 — Supplementary Information [file 41467_2019_12250_MOESM1_ESM.pdf]

Supplementary Information

Molecular mechanism of azoxy bond formation for azoxymycins biosynthesis

Guo *et al*.

Supplementary Figures

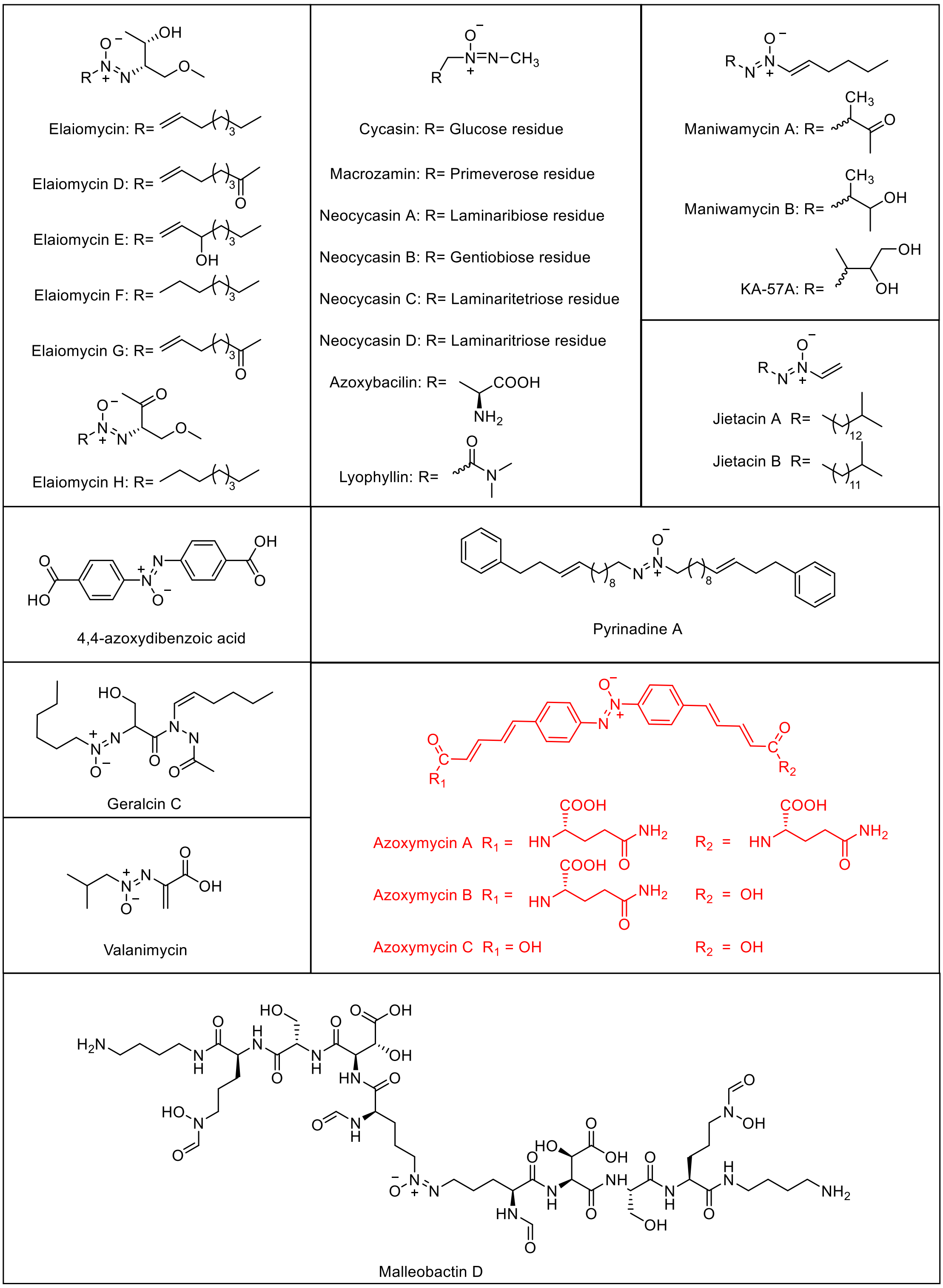

Supplementary Figure 1: Structures of azoxy natural products (azoxymycins are highlighted in red)[1-9].

```
AzoC  1    MSSRAPEELTGVQSpELPAYDPDDQAENAVIARLAGNWHRRAAV--KREEPNLADLFELARDDYPERILPFRDHPTFRAL  78

AurF  1    MREEQPHLATTWAA---RGWVEEEGIGSATLGRLVRAWPRRAAVVNKADILDEWADYDTLVPDYPLEIVPFAEHPLFLAA  77
CmlI  1    ---------------HHHHHHHHAIAENAVINRLVGNWHRRAAV--KREEPDVYALFDPGRPDFREDMIPFRGHPIWERL  63


AzoC  79   PPEDRARLLSWAWISYNRTTVLLEGQIVNPAFQLGLDGEFPQPVSELMQRSLAQAMVDEQYHTLMHLNASAVTRRRRGEA  158
AurF  78   EPHQRQRVLTGMWIGYNERVIATEQLIAEPAFDLVMHGVFPGSDDPLIRKSVQQAIVDESFHTYMHMLAIDRTRELRK--  155
CmlI  64   SDETRSRLLSWGWVAYNRNTVLIEQRIANPAFELVIGGAYPGLGGQQLELAVAQAMVDEQYHTLMHINGSAVTRRMRRSD  143


AzoC  159  FADAALPKPLVVREHEAR--LASCANERERRLTTLAFATVAEISINAYLNLIADDKEIQPVNSATVRIHNRDEYCHASIS  236
AurF  156  ISERP-PQPELVTYRRLRRVLADMPEQWERDIAVLVWGAVAETCINALLALLARDATIQPMHSLITTLHLRDETAHGSIV  234
CmlI  144  FSDRVLPDSHITTIHQEH--LDRCEEPWQRSLTTLGFATVAEISINAYLDLLADDQEIQVVNSTTVKLHNRDEYCHASIS  221


AzoC  237  AVLAEQVHHTLDDGERRYFLQSLVAGLEAFVGNDFMAWHRIMDEAGIRGGHEMLDDIQHAGGRKRLVQDFSGLRKLVERL  316
AurF  235  VEVVRELYARMNEQQRRALVRCLPIALEAFAEQDLSALLLELNAAGIRGAEEIVGDLRSTAGGTRLVRDFSGARKMVEQL  314
CmlI  222  GEMMKQVYEALPADRRRFLLEKVVAGLEAFVAPDFTTWESIVAFEGVPGWEKAAAEVREAQGGTHLVQDHSGIHTLLTEM  301


AzoC  317  DAVDDLDFDWSRSVTGSDAVSPTR  340
AurF  315  GLDDAVDFDFPERPDWSPHTP--R  336
CmlI  302  DVLDQVEFGWGTTVT--------R  317
```

Supplementary Figure 2: Sequence alignment of AzoC (accession number: AKQ24642), AurF (accession number: CAE02601) and CmlI (accession number: 5HYH_A) using COBALT (https://www.ncbi.nlm.nih.gov/tools/cobalt). Iron binding motifs are highlighted in yellow, and conserved residues are marked in red.

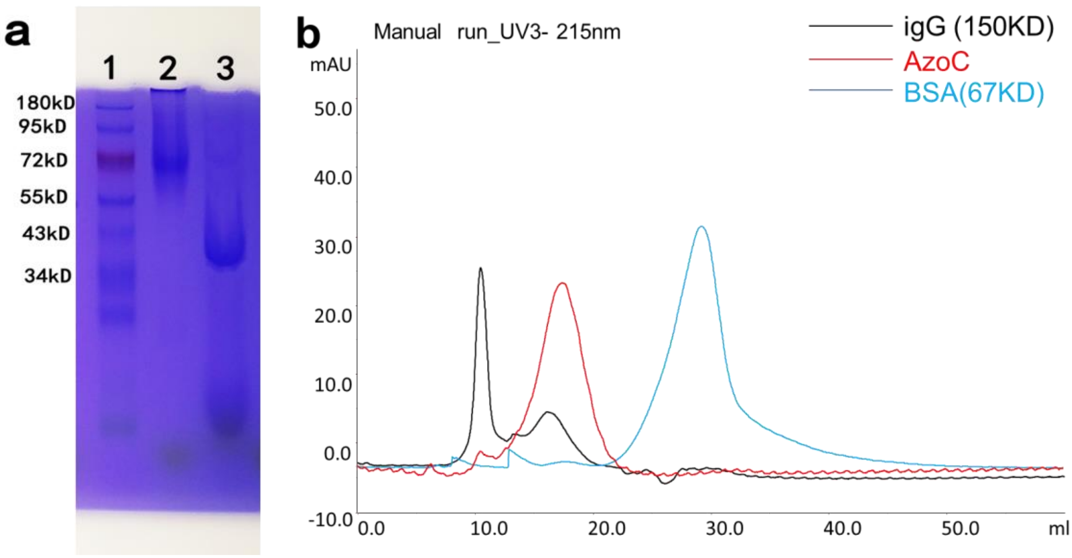

Supplementary Figure 3: Analysis of purified AzoC. **a** Native-PAGE analysis of purified AzoC. Lane 1, protein marker; 2, AzoC; 3, boiled AzoC. Theoretical molecular weight of recombinant AzoC monomer was 39 KD. The measured molecular weight of denatured AzoC was between 34 KD and 43KD (Lane 3), and the measured molecular weight of AzoC was approximately 72 KD (Source data are provided as a Source Data file). **b** Size exclusion chromatography of purified AzoC. Molecular weight marker proteins are shown in black (IgG 150 KD) and blue (BSA 67 KD), and AzoC is shown in red. The result suggested that molecular weight of AzoC in solution is between 67 KD and 150 KD.

a

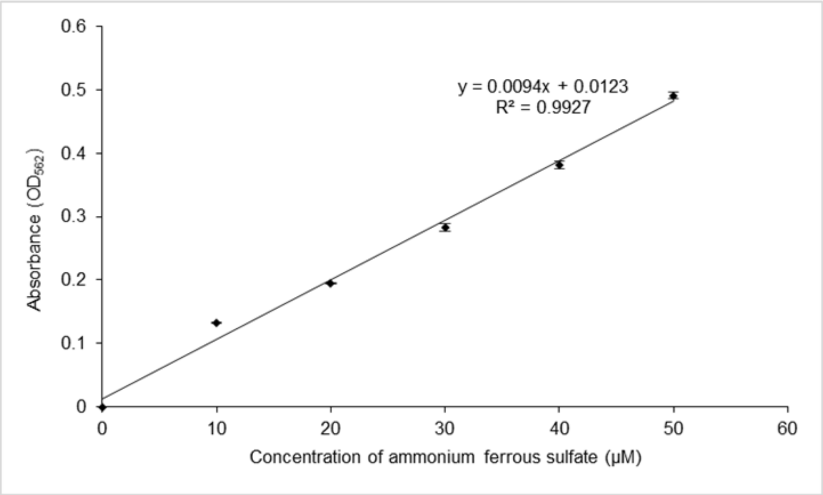

b

	Sample1	Sample2	Sample3	Standard deviation	Average	Iron/enzyme ratio
Absorbance(OD_{562})	0.348	0.353	0.339	\	0.347	\
Iron concentration of AzoC (μM)	35.713	36.245	34.755	0.616	35.571	1.779

Supplementary Figure 4: Determination iron to enzyme ratio of AzoC with Ferrozine assay. **a** Ammonium ferrous sulfate was used to prepare the standard solutions, and molar extinction coefficient constant at 562 nm was 28,000 M^{-1} cm^{-1}. Each data point was measured in triplicate. Error bars indicate one standard deviation. **b** Iron concentration of AzoC assayed with spectrophotometer. Protein concentration of AzoC was 20 μ M (Source data are provided as a Source Data file).

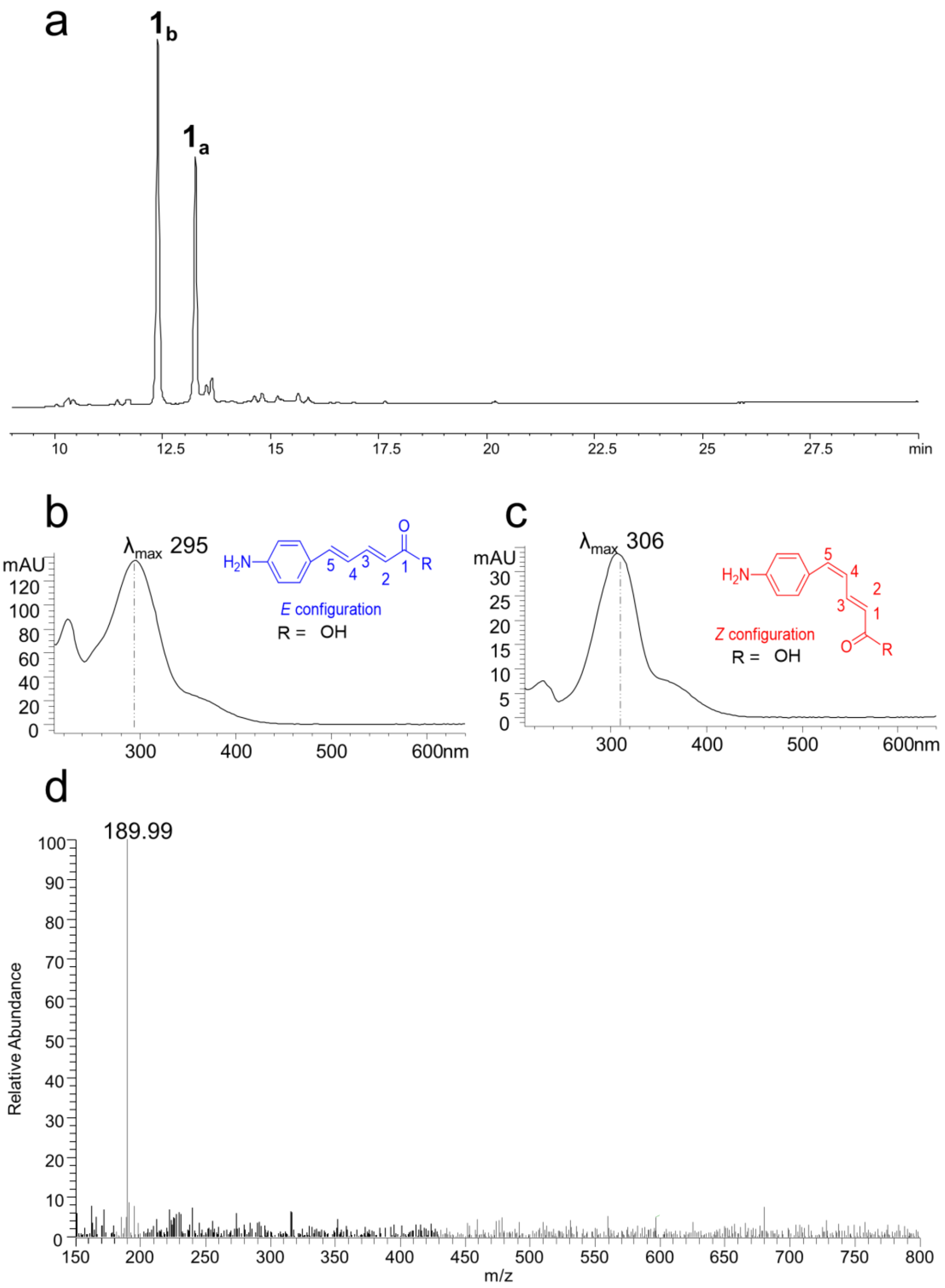

Supplementary Figure 5: Characterization of purified **1**. **a** HPLC analysis of **1** at 370 nm, $\mathbf{1_a}$ and $\mathbf{1_b}$ are the C4 *Z*/*E* isomers respectively. **b** UV/vis spectrum of $\mathbf{1_a}$. **c** UV/vis spectrum of $\mathbf{1_b}$. **d** MS of **1** ($[M+H]^+$ 189.99).

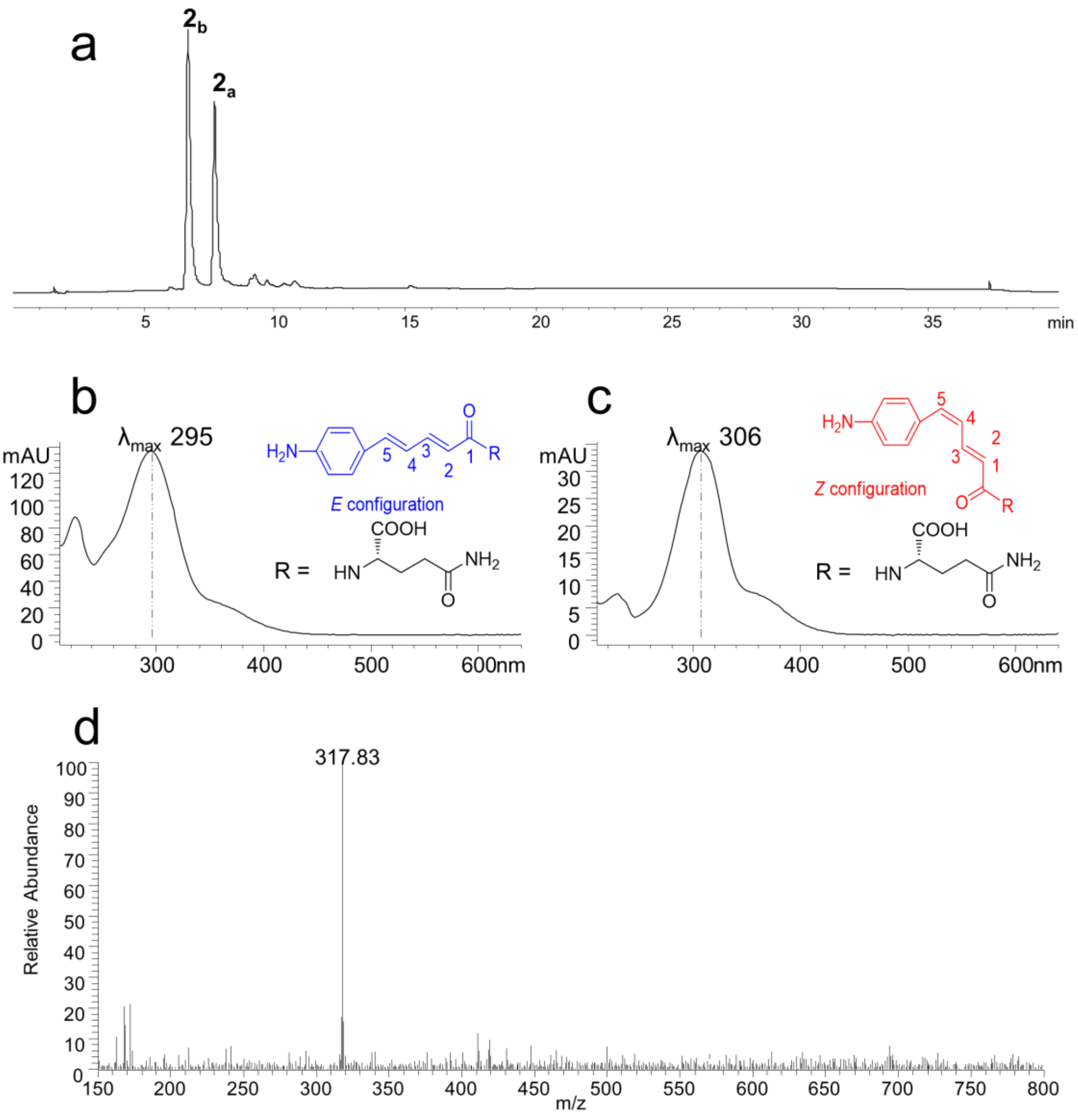

Supplementary Figure 6: Characterization of purified **2**. **a** HPLC analysis of **2** at 370 nm, $\mathbf{2_a}$ and $\mathbf{2_b}$ are the C4 *Z*/*E* isomers respectively. **b** UV/vis spectrum of $\mathbf{2_a}$. **c** UV/vis spectrum of $\mathbf{2_b}$. **d** MS of **2** ($[M+H]^+$ 317.83).

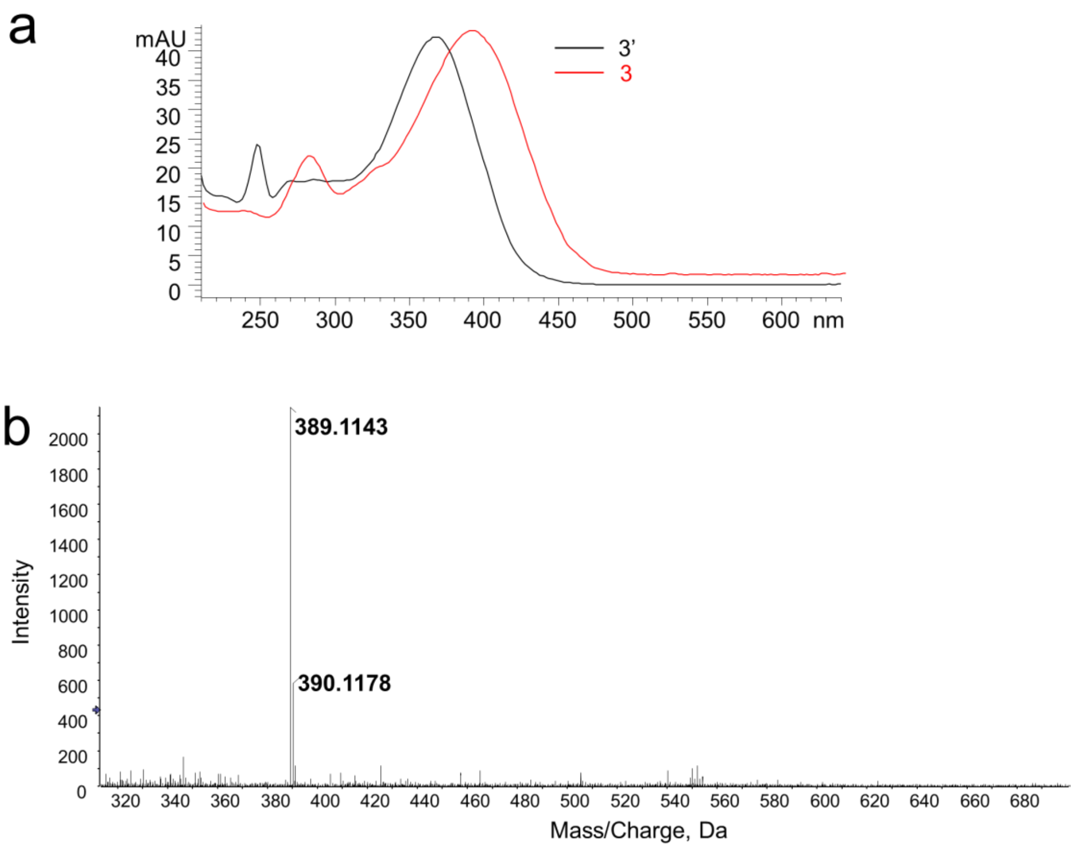

Supplementary Figure 7: Characterization of purified **3** and **3'**. **a** UV/vis spectra of **3'** (black curve) and **3** (red curve). **b**HRMS of **3'**, $[M-H]^-$ 389.1143, molecular formula is calculated as $C_{22}H_{18}N_2O_5$.

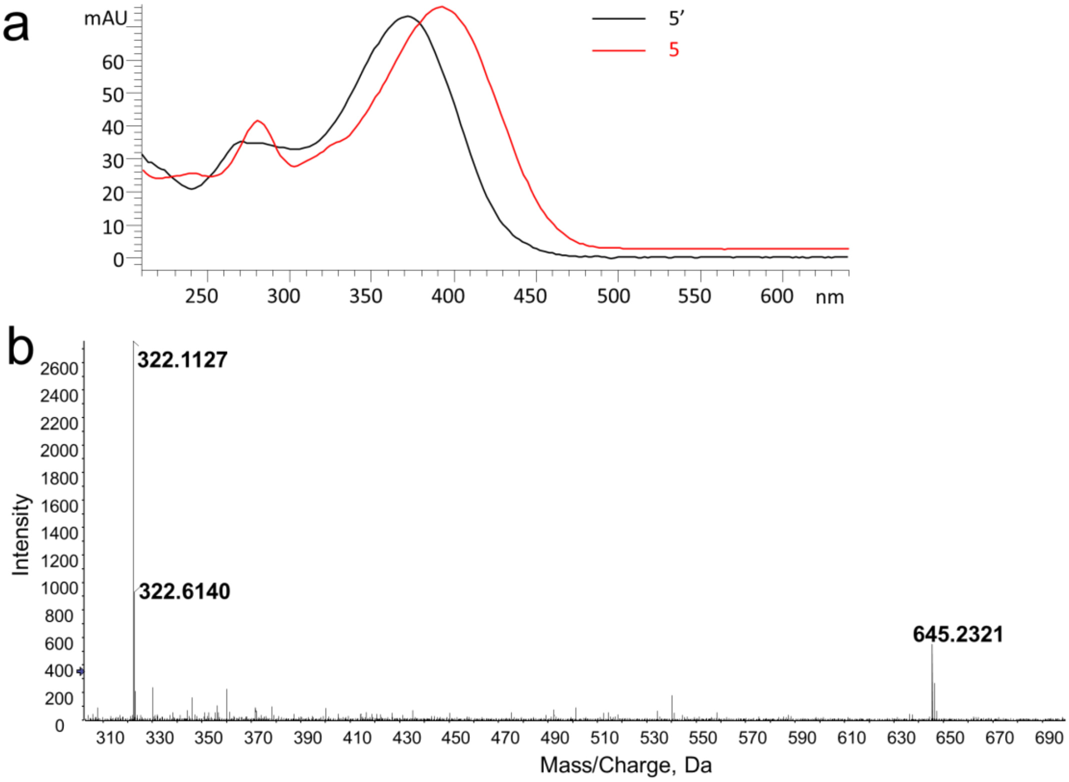

Supplementary Figure 8: Characterization of purified **5** and **5’**.**a** Uv/vis spectra of **5’** (black curve) and **5** (red curve). **b** HRMS of **5’**, $[M-H]^-$ 645.2321, $[M-2H]^{2-}$ 322.1127. The molecular formula was calculated as $C_{32}H_{34}N_6O_9$.

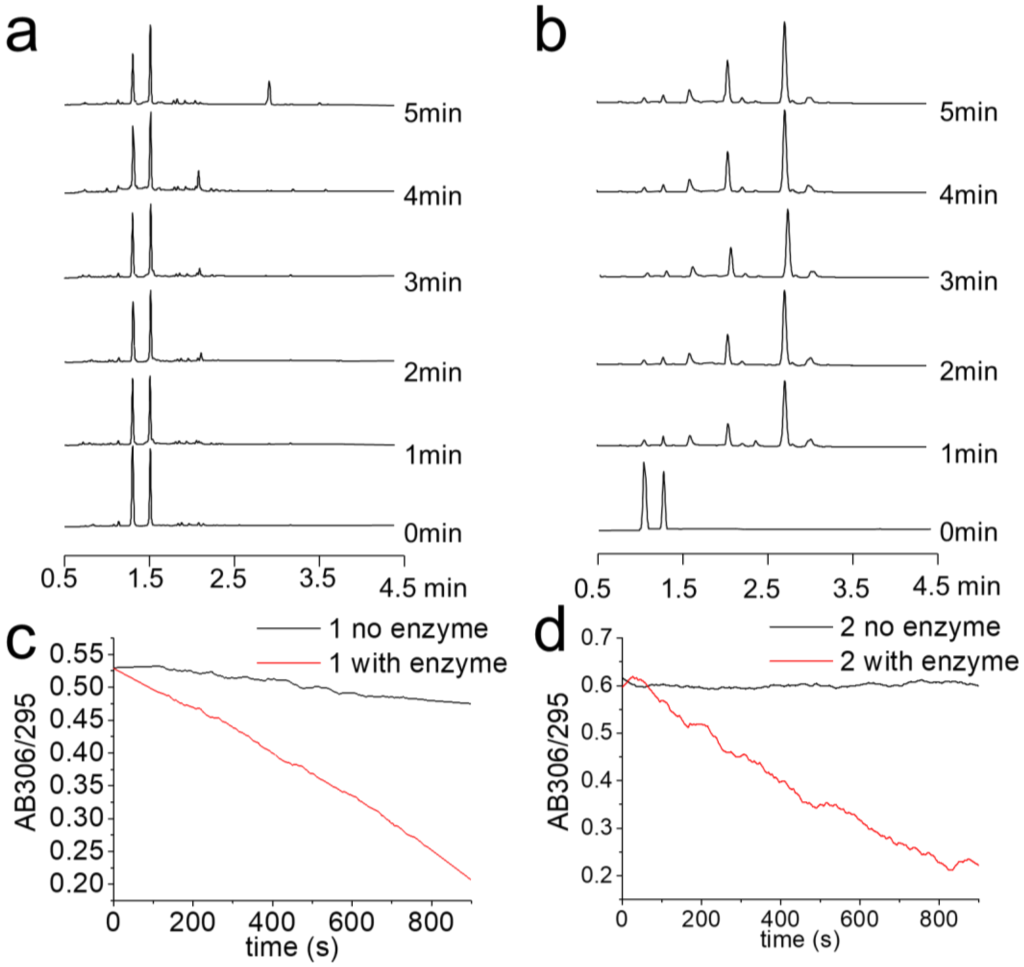

Supplementary Figure 9: Isomers consumption of **1** and **2** by AzoC. **a** Fast HPLC analysis of $\mathbf{1_a}$ and $\mathbf{1_b}$'s consumption at 370 nm. The time-course analysis indicated that $\mathbf{1_b}$ decreased faster than $\mathbf{1_a}$ during the reaction process. **b** Fast HPLC analysis of $\mathbf{2_a}$ and $\mathbf{2_b}$'s consumption at 370 nm. The time-course analysis indicated that $\mathbf{2_b}$ decreased faster than $\mathbf{2_a}$ during the reaction process. **c** Isomers consuming dynamic curves of $\mathbf{1_a}$ and $\mathbf{1_b}$ assayed with spectrophotometer. 306 nm is the maximal absorbance of the *Z* isomer ($\mathbf{1_b}$), 295 nm is the maximal absorbance of the *E* isomer ($\mathbf{1_a}$). Without the addition of AzoC, the absorbance ratio of 306 nm to 295 nm was stable, indicating that isomers were in balance. While with the addition of AzoC, the absorbance ration of 306 nm to 295 nm was decreased, indicating that $\mathbf{1_b}$ is consumed faster than $\mathbf{1_a}$. **d** Isomers consuming dynamic curves of $\mathbf{2_a}$ and $\mathbf{2_b}$ assayed with spectrophotometer. 306 nm is the maximal absorbance of *Z* isomer ($\mathbf{2_b}$), 295 nm is the maximal absorbance of *E* isomer ($\mathbf{2_a}$). Without the addition of AzoC, the absorbance ratio of 306 nm to 295 nm was stable, indicating that isomers were in balance. While with the addition of AzoC, the absorbance ration of 306 nm to 295 nm was decreased, indicating that $\mathbf{2_b}$ is consumed faster than $\mathbf{2_a}$.

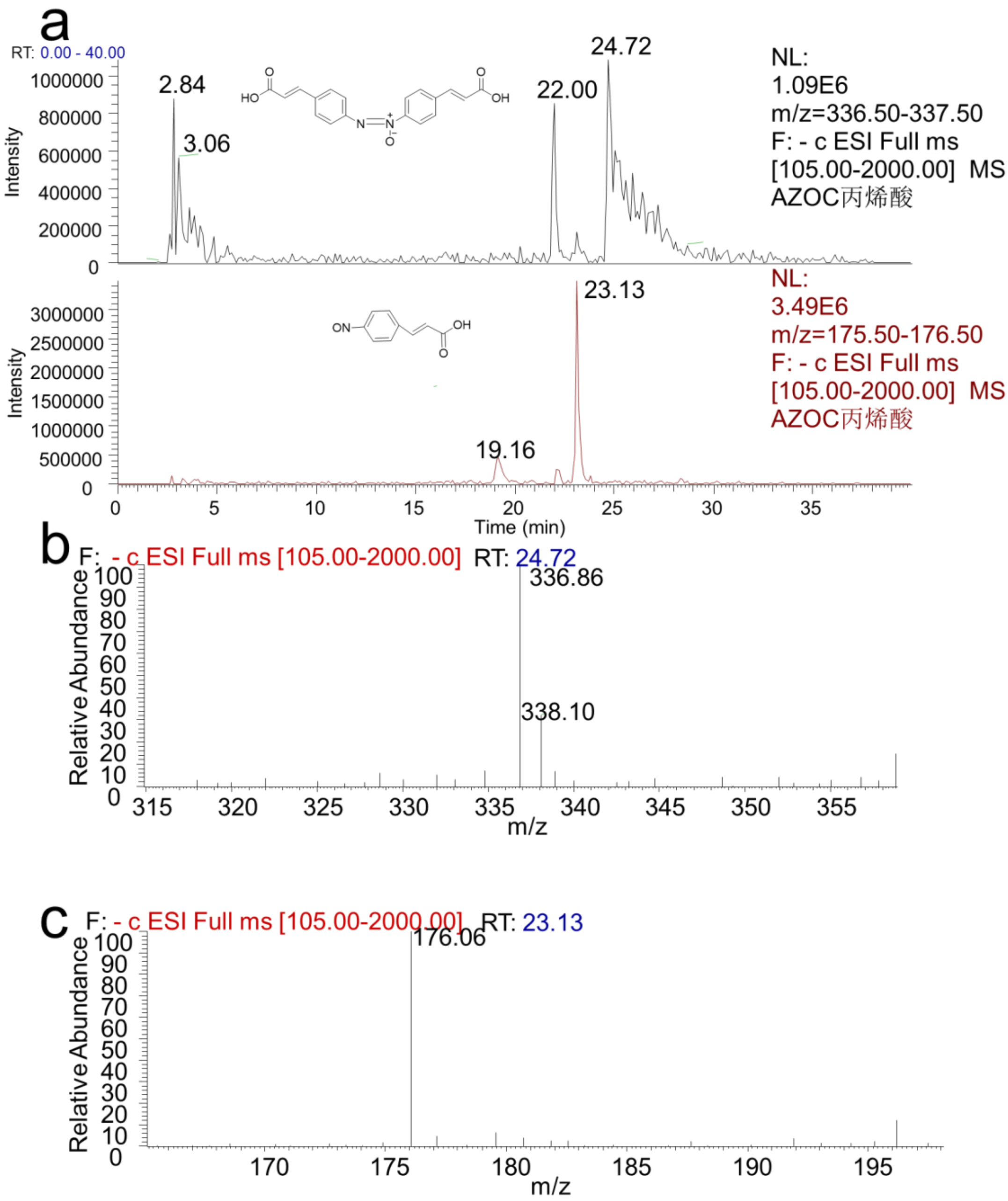

Supplementary Figure 10: LC-MS analysis of **6**'s *in vitro* reaction with AzoC. Reaction was performed by incubating 100 μM **6** with 10 μM AzoC, 1 mM NADH, 10 μM phenazine methosulfate (PMS) in 20 mM HEPES buffer (pH 7) at 30 ℃ for 20 min. Then the mixture was subject to LC-MS analysis. **a** Extracted ion chromatogram (EIC) of the reaction mixture (azoxy product *m/z* 337, nitroso product *m/z* 176, $[M-H]^-$). **b** MS at RT 24.72 min ($[M-H]^-$ 336.86). **c** MS at RT 23.13 min ($[M-H]^-$ 176.06)

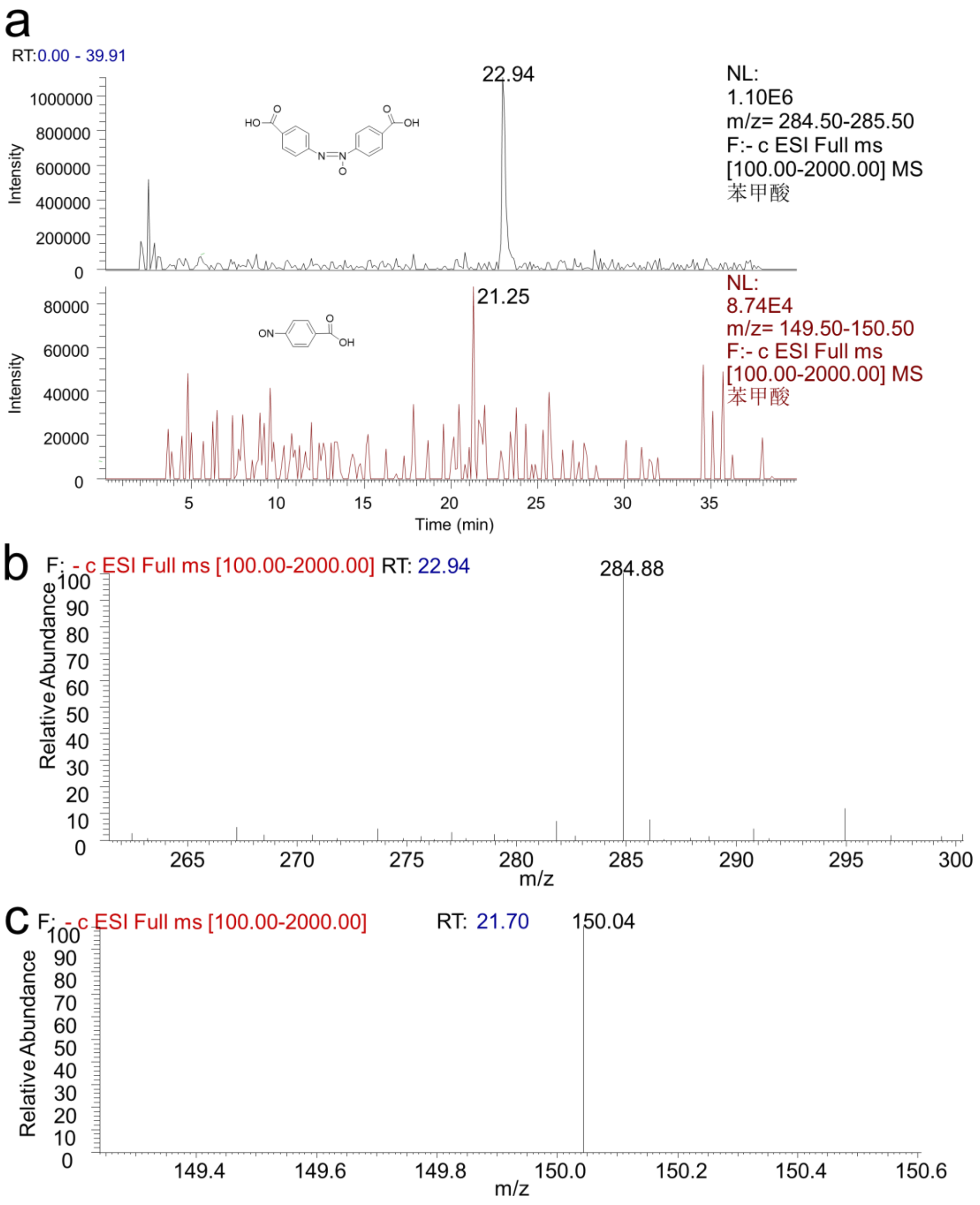

Supplementary Figure 11: LC-MS analysis of **11**'s *in vitro* experiments. Reaction was performed by incubating 100 μM **11** with 10 μM AzoC, 1 mM NADH, 10 μM phenazine methosulfate (PMS) in 20 mM HEPES buffer (pH 7) at 30℃ for 20 min. Then the mixture was subject to LC-MS analysis. **a** Extracted ion chromatogram (EIC) of the reaction mixture (azoxy product *m/z* 285, nitroso product *m/z* 150, $[M-H]^-$). **b** MS at RT 22.94 min ($[M-H]^-$ 284.88). **c** MS at RT 21.70 min ($[M-H]^-$ 150.04)

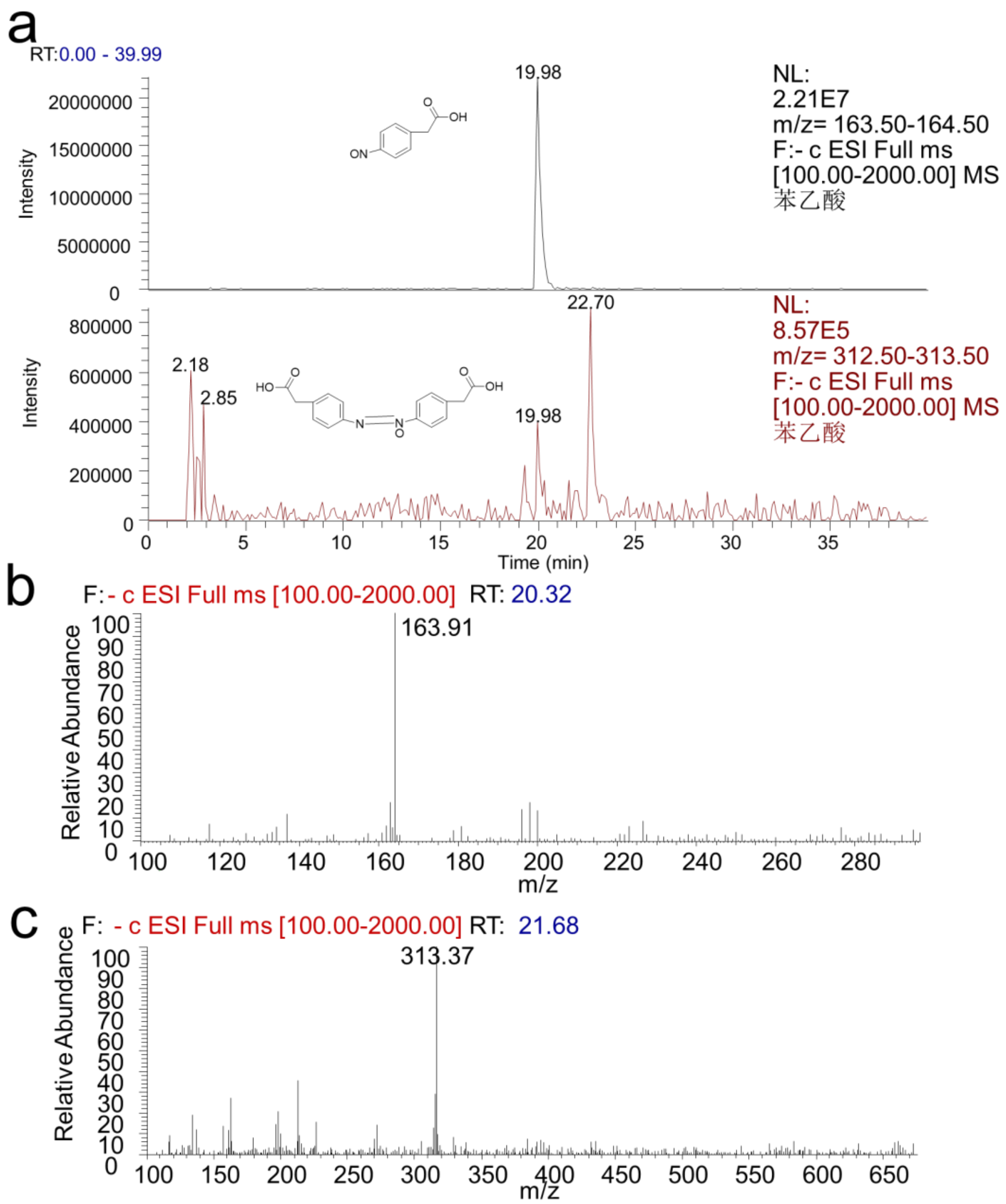

Supplementary Figure 12: LC-MS analysis of **12**'s *in vitro* experiments. Reaction was performed by incubating 100 μM **12** with 10 μM AzoC, 1 mM NADH, 10 μM phenazine methosulfate (PMS) in 20 mM HEPES buffer (pH 7) at 30℃ for 20 min. Then the mixture was subject to LC-MS analysis. **a** Extracted ion chromatogram (EIC) of the reaction mixture (azoxy product *m/z* 164, nitroso product *m/z* 313, $[M-H]^-$). **b** MS at RT 20.32 min ($[M-H]^-$ 163.91). **c** MS at RT 21.68 min ($[M-H]^-$ 313.37)

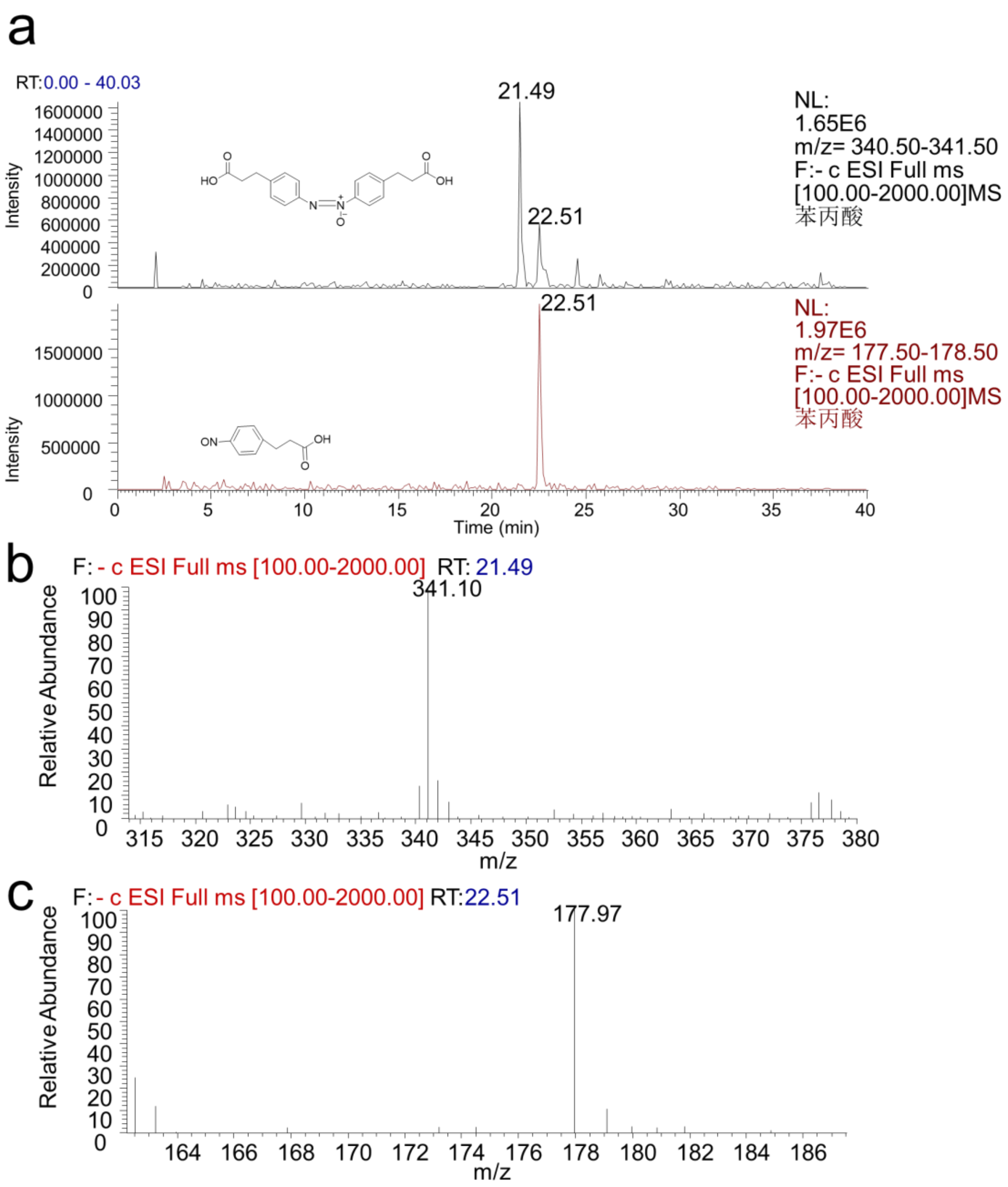

Supplementary Figure 13: LC-MS analysis of **13**'s *in vitro* experiments. Reaction was performed by incubating 100 μM **13** with 10 μM AzoC, 1 mM NADH, 10 μM phenazine methosulfate (PMS) in 20 mM HEPES buffer (pH 7) at 30℃ for 20 min. Then the mixture was subject to LC-MS analysis. **a** Extracted ion chromatogram (EIC) of the reaction mixture (azoxy product *m/z* 341, nitroso product *m/z* 178, $[M-H]^-$). **b** MS at RT 21.49 min ($[M-H]^-$ 341.10). **c** MS at RT 22.51 min ($[M-H]^-$ 177.97)

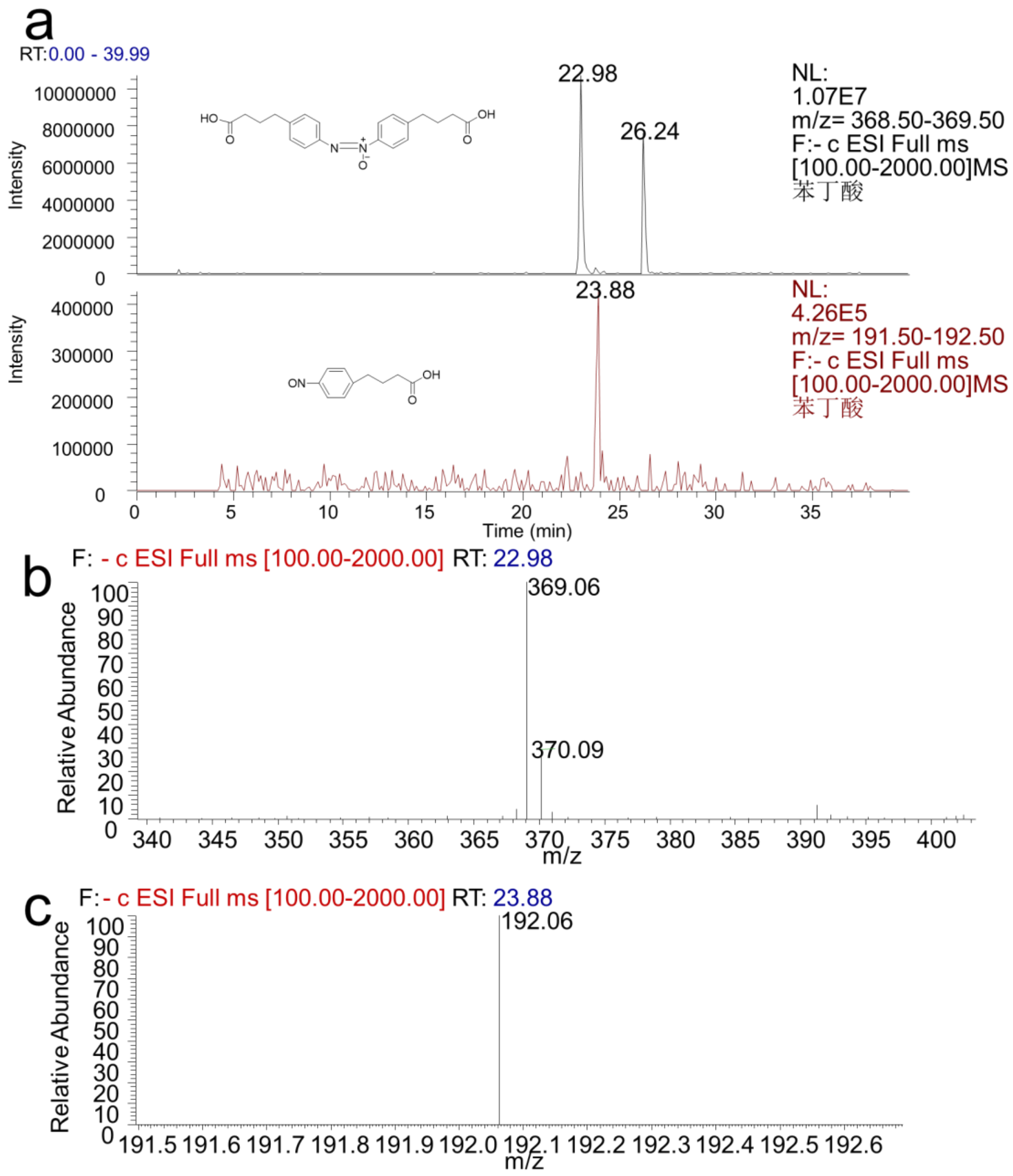

Supplementary Figure 14: LC-MS analysis of **14**'s *in vitro* experiments. Reaction was performed by incubating 100 μM **14** with 10 μM AzoC, 1 mM NADH, 10 μM phenazine methosulfate (PMS) in 20 mM HEPES buffer (pH 7)at 30 ℃ for 20 min. Then the mixture was subject to LC-MS analysis. **a** Extracted ion chromatogram (EIC) of the reaction mixture (azoxy product *m/z* 369, nitroso product *m/z* 192, $[M-H]^-$). **b** MS at RT 22.98 min ($[M-H]^-$ 369.06). **c** MS at RT 23.88 min ($[M-H]^-$ 192.06)

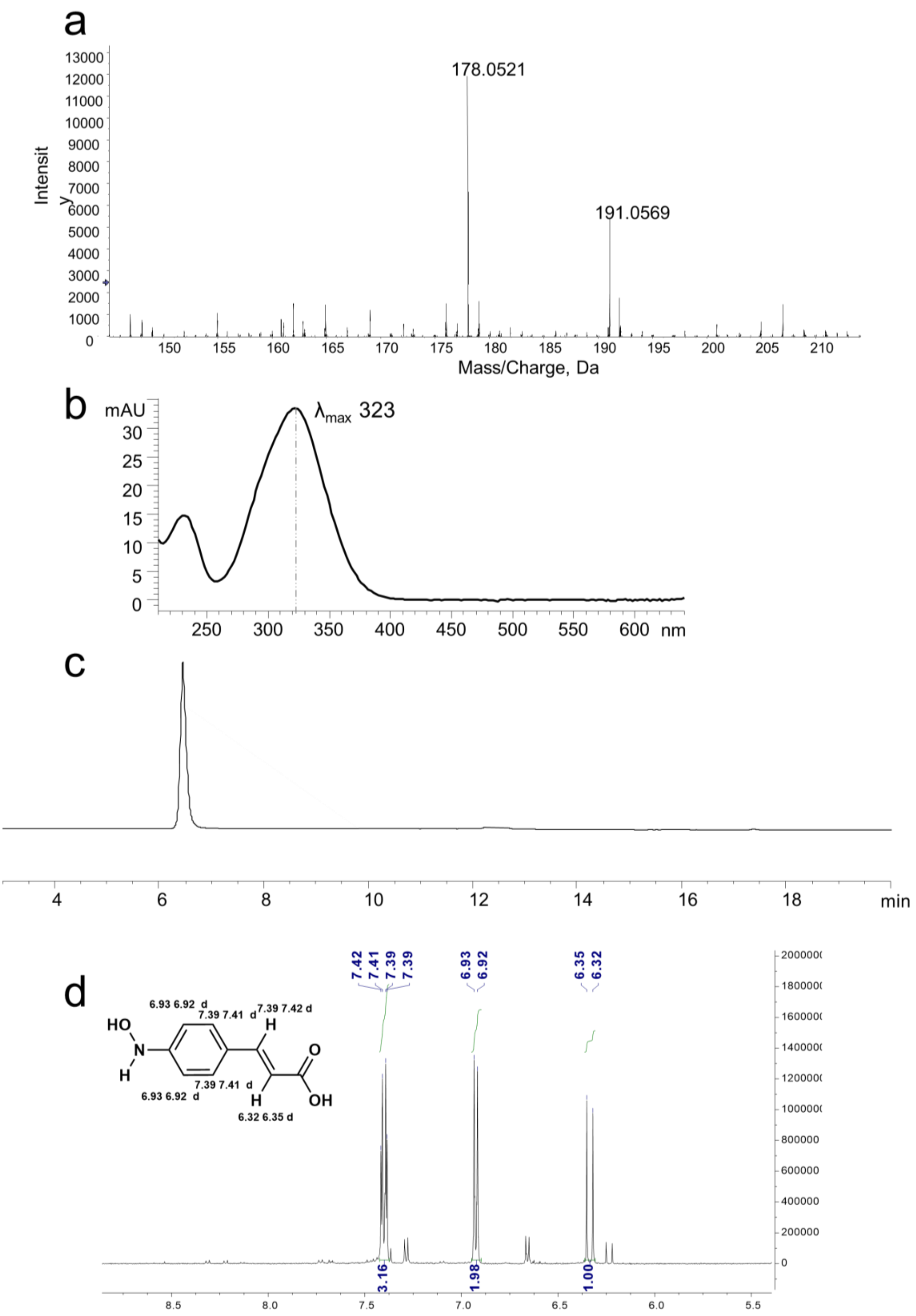

Supplementary Figure 15: Identification of synthesized **7. a** HRMS of synthesized **7** ($[M\text{-}H]^-$ 178.0521, molecular formula is calculated as $C_9H_9NO_3$). **b** UV/vis spectrum of synthesized **7**. **c** HPLC analysis of synthesized **7** at 370 nm. **d** ^{1}H NMR of synthesized **7** in Methanol-*d*4. (Note: hydroxylamine arene is very active during the purification process and influence the NMR purity.)

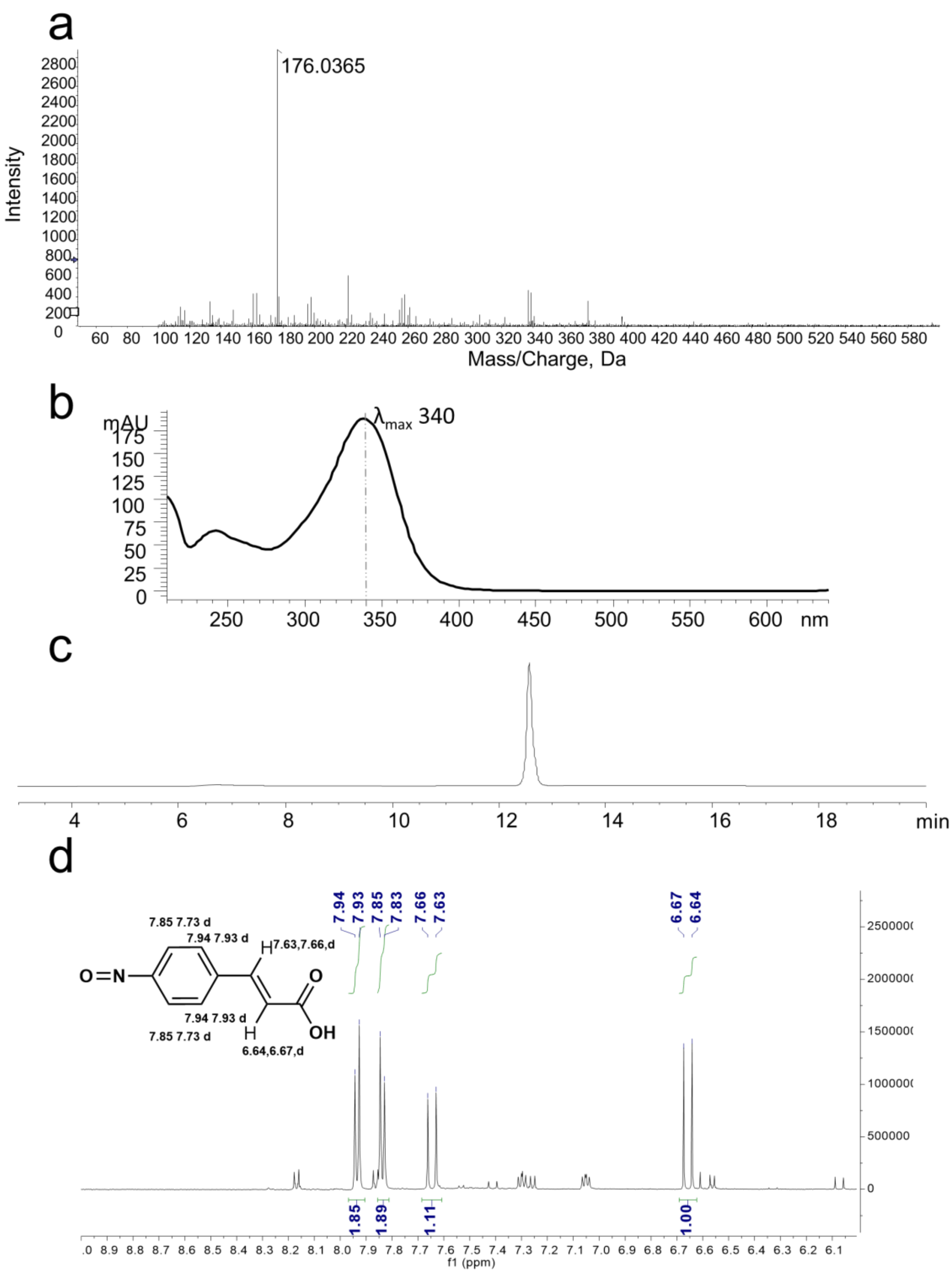

Supplementary Figure 16: Identification of synthesized **8. a** HRMS of synthesized **8** ($[M-H]^-$ 176.0365, molecular formula is calculated as $C_9H_7NO_3$). **b** UV/vis spectrum of synthesized **8**. **c** HPLC analysis of synthesized **8** at 370 nm.**d** ^{1}H NMR of synthesized **8** in Acetone-*d*6. (Note: nitroso arene is very active during the purification process and influence the NMR purity.)

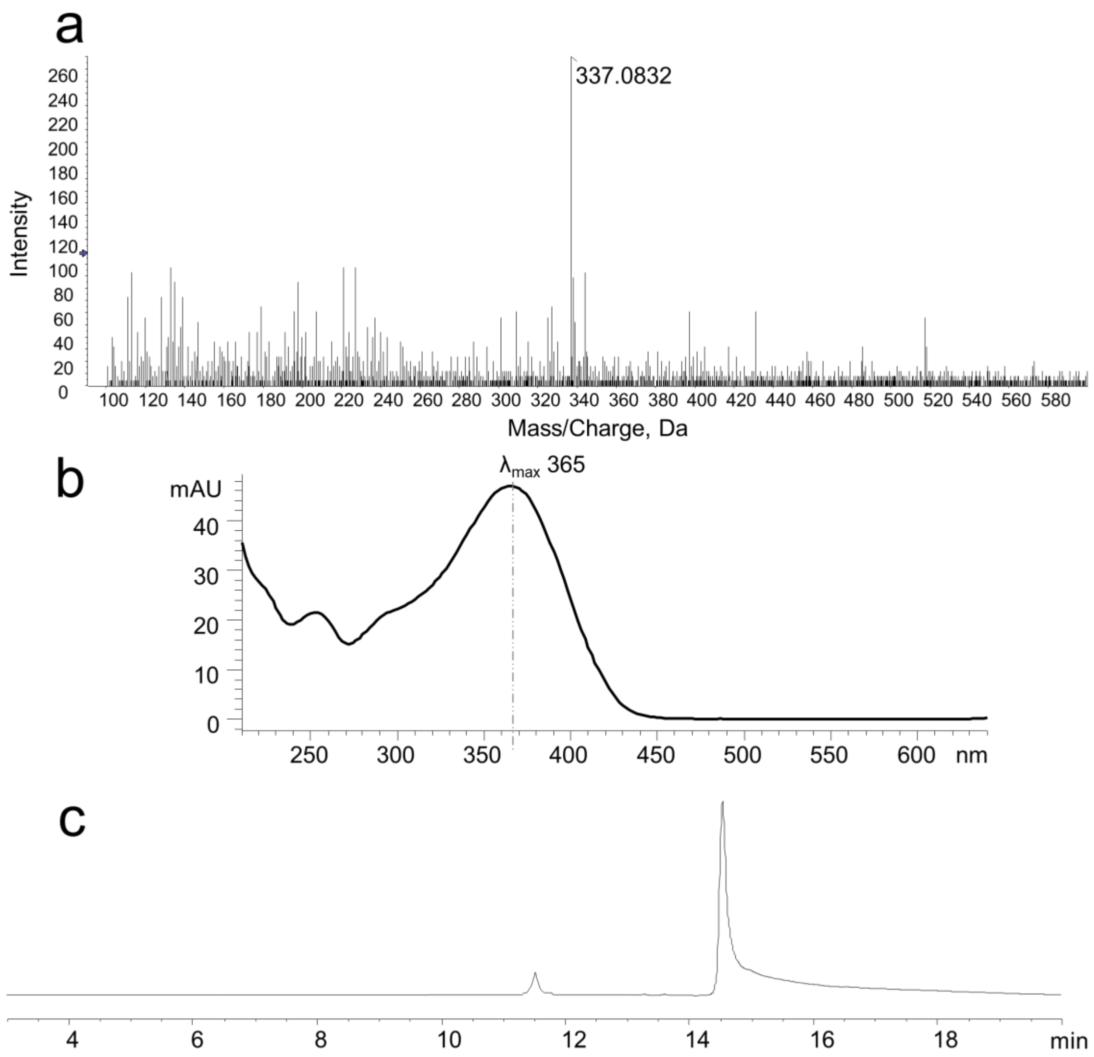

Supplementary Figure 17: Identification of synthesized **9. a** HRMS of synthesized **9** ($[M\text{-}H]^-$ 337.0832, molecular formula is calculated as $C_{18}H_{14}N_2O_5$). **b** UV/vi*s* spectrum of synthesized **9**. **c** HPLC analysis of synthesized **9** at 370 nm.

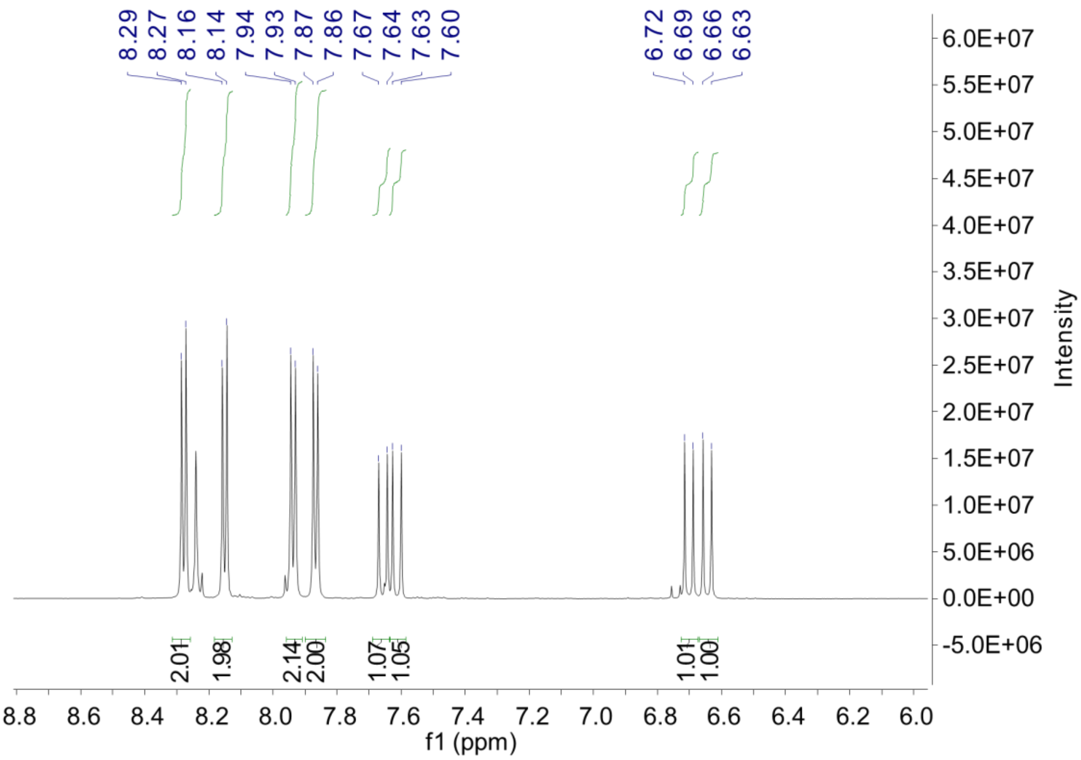

Supplementary Figure 18: ^{1}H-NMR of synthesized **9** in DMSO-*d*6.

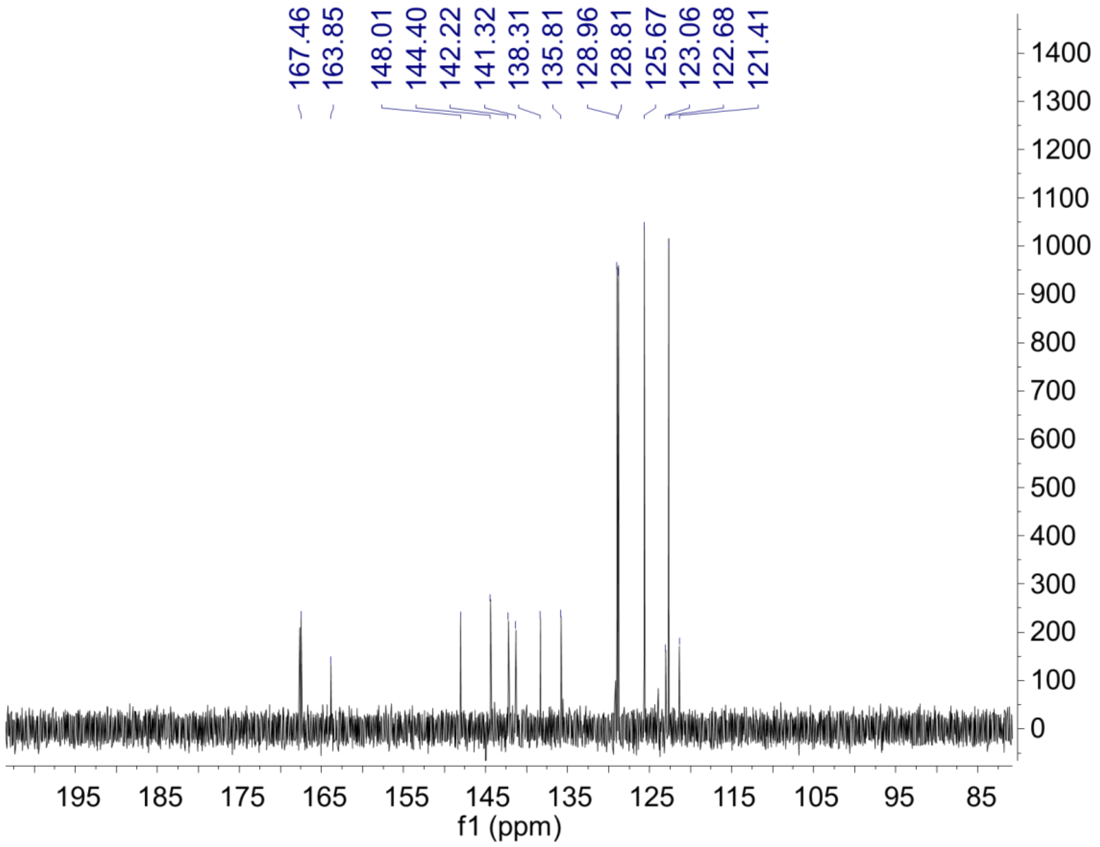

Supplementary Figure 19: ^{13}C-NMR of synthesized **9** in DMSO-*d*6**.**

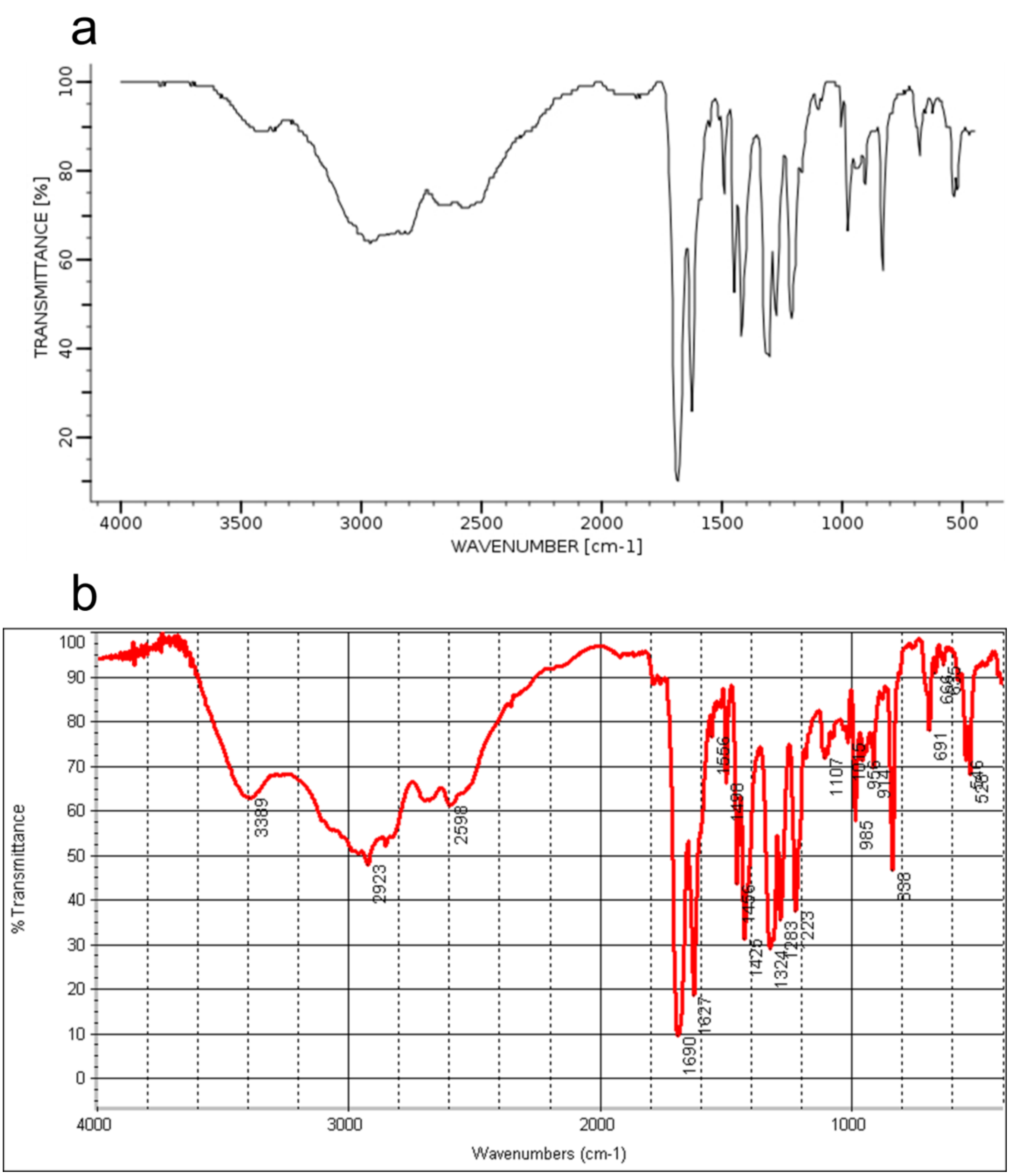

Supplementary Figure 20: Infrared spectra of synthesized **9** .**a** Infrared spectrum of azoxy phenyl propenoic acid in Bio-Rad Laboratories data base. **b** Infrared spectrum of synthesized **9**.

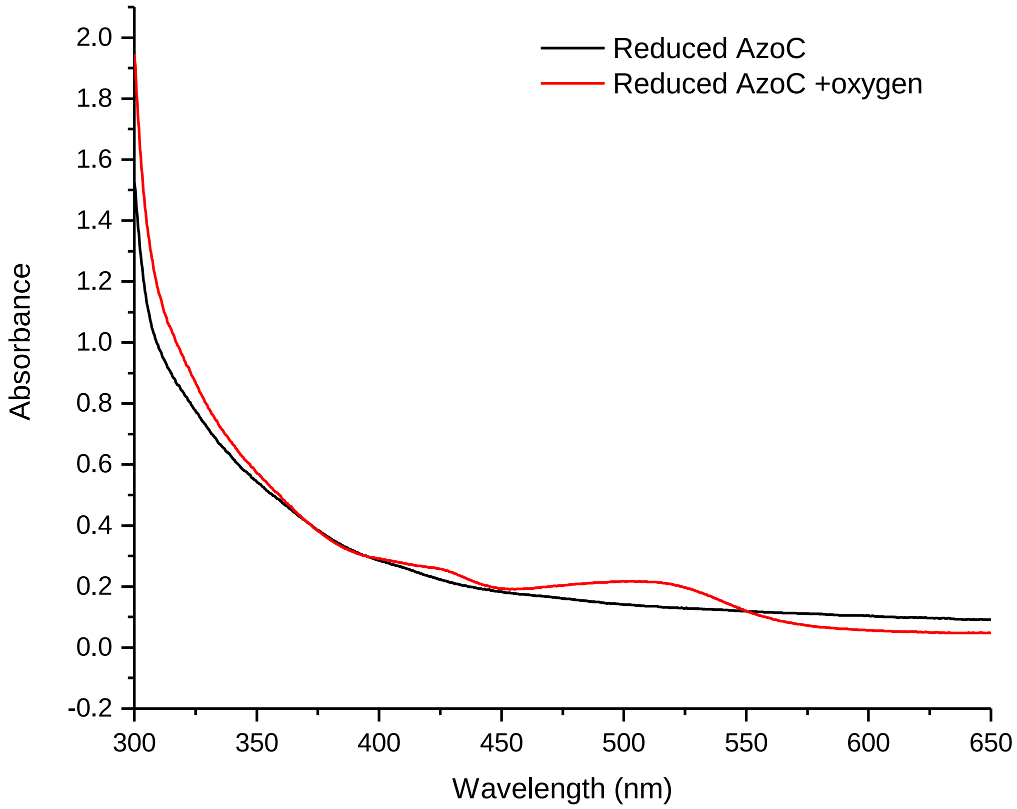

Supplementary Figure 21: Optical absorption spectra of reduced AzoC (2 mM) before and after exposed to oxygen. Buffer: 20 mM HEPES, pH 7. AzoC was pre-reduced by PMS and ascorbic acid and washed with deoxygenated buffer for three times. The black line is reduced AzoC, and the red line is the reduced AzoC after exposing to oxygen.

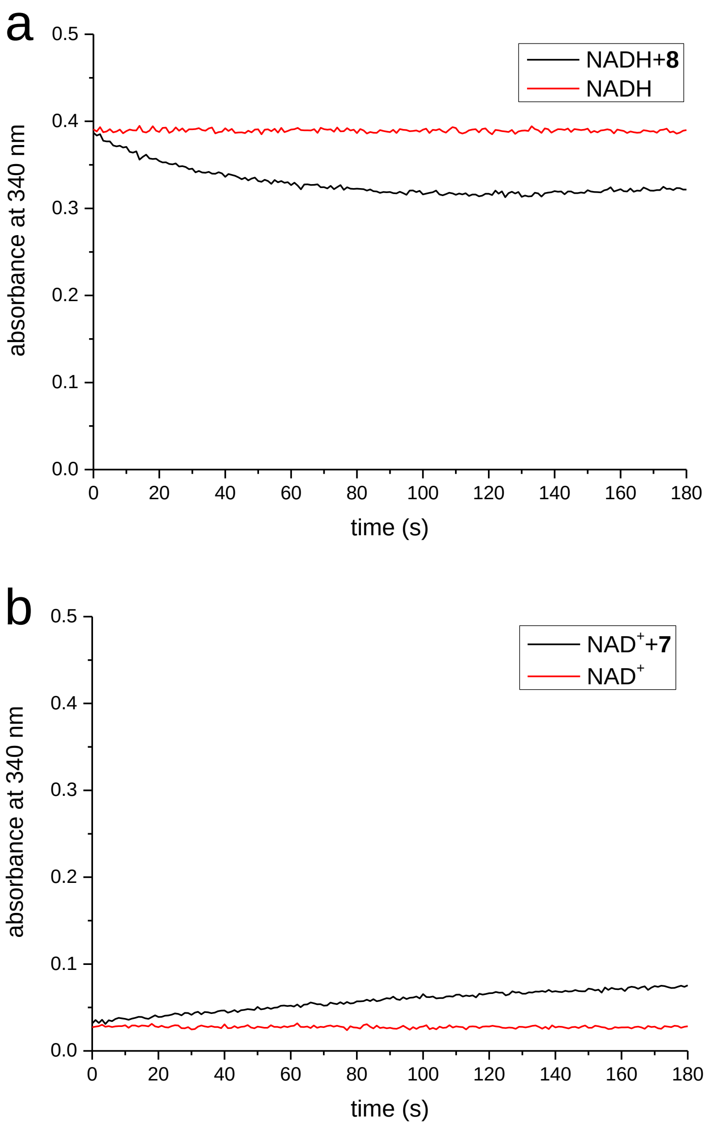

Supplementary Figure 22: NAD^+ and NADH changing dynamics when incubated with **8** or **7**. The experiments was assayed by measuring the absorbance at 340 nm (340 nm is the characteristic absorbance peak of NADH) with spectrophotometer. **a** NADH changing dynamics when reducing **8**. The black line was the NADH consuming curve when 100 μ M **8** was incubated with 10 eq NADH, during the reaction process NADH concentration reduced about 16% within 60 s and kept stable. The red line was the control example (NADH without **8**). **b** NAD^+ changing dynamics when oxidizing **7**. The black line was the NAD^+ changing curve when 100 μ M **7** was incubated with 10 eq NAD^+ (the characteristic absorbance peak of NAD^+ was 260 nm, which was vulnerable to be confused with reagents absorbance within the reaction system, hence 340 nm of NADH absorbance was employed to reflect NAD^+ changing), during the reaction process about 10~12% NAD^+ was converted to NADH within the 180 s reaction process. The red line was the control example (NAD^+ without **7**).

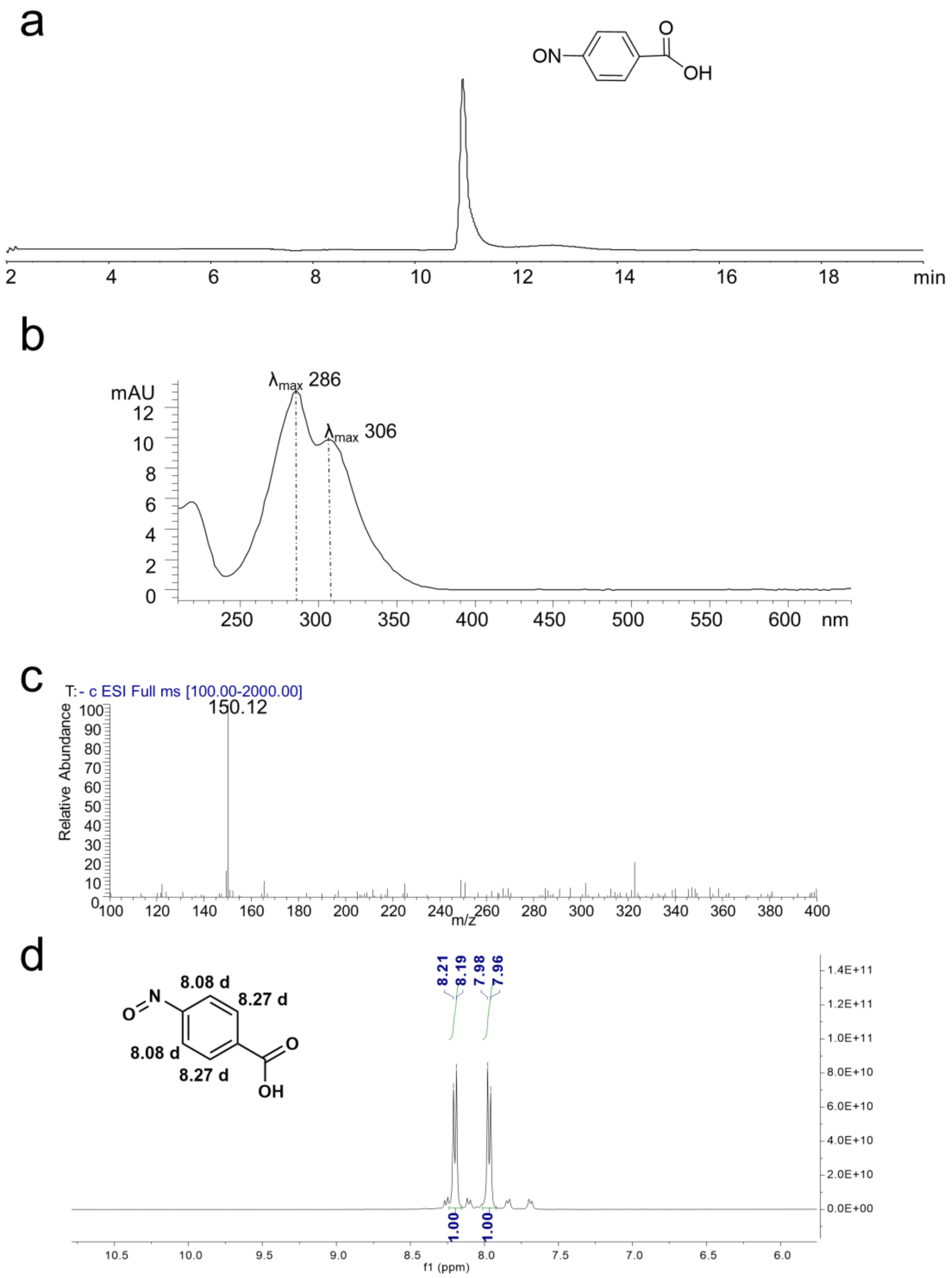

Supplementary Figure 23: Identification of synthesized *p*-nitroso benzoic acid. **a** HPLC analysis of *p*-nitroso benzoic acid at 370 nm. Retention time was 11.13 min. **b** UV/vis spectrum of *p*-nitroso benzoic acid. **(c)** MS of *p*-nitroso benzoic acid ([M-H]$^-$ 150.12). **d** ^{1}H NMR of synthesized *p*-nitroso benzoic acid in DMSO-*d*6. (Note: nitroso arene is very active during the purification process and influence the NMR purity.)

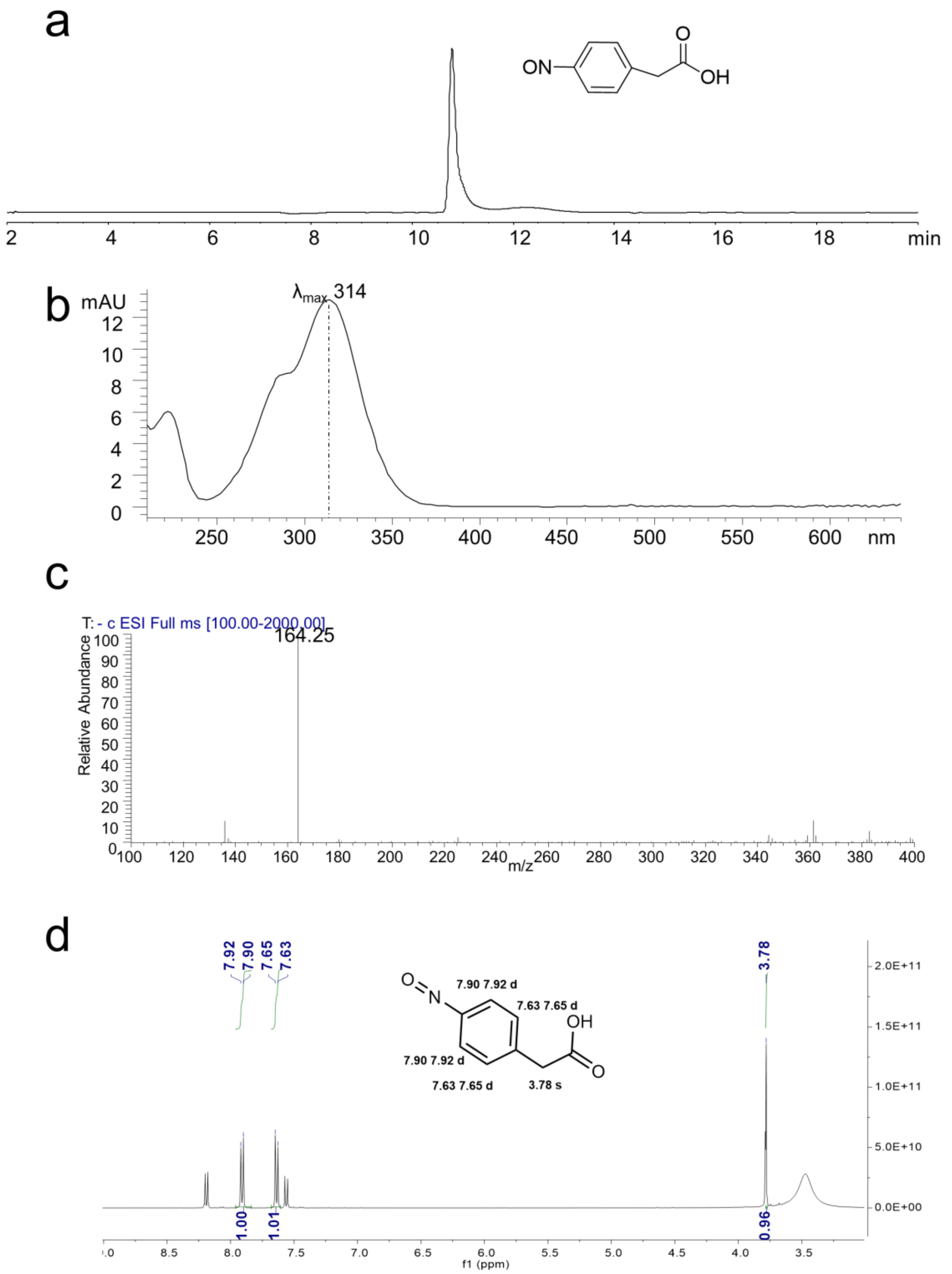

Supplementary Figure 24: Identification of synthesized *p*-nitroso phenylacetic acid. **a** HPLC analysis of *p*-nitroso phenylacetic acid at 370 nm. Retention time was 10.76 min. **b** UV/vis spectrum of *p*-nitroso phenylacetic acid. **c** MS of *p*-nitroso phenylacetic acid ($[M-H]^-$ 164.25). **d** ^1H NMR of synthesized *p*-nitroso phenylacetic acid in acetone-*d*6. (Note: nitroso arene is very active during the purification process and influence the NMR purity.)

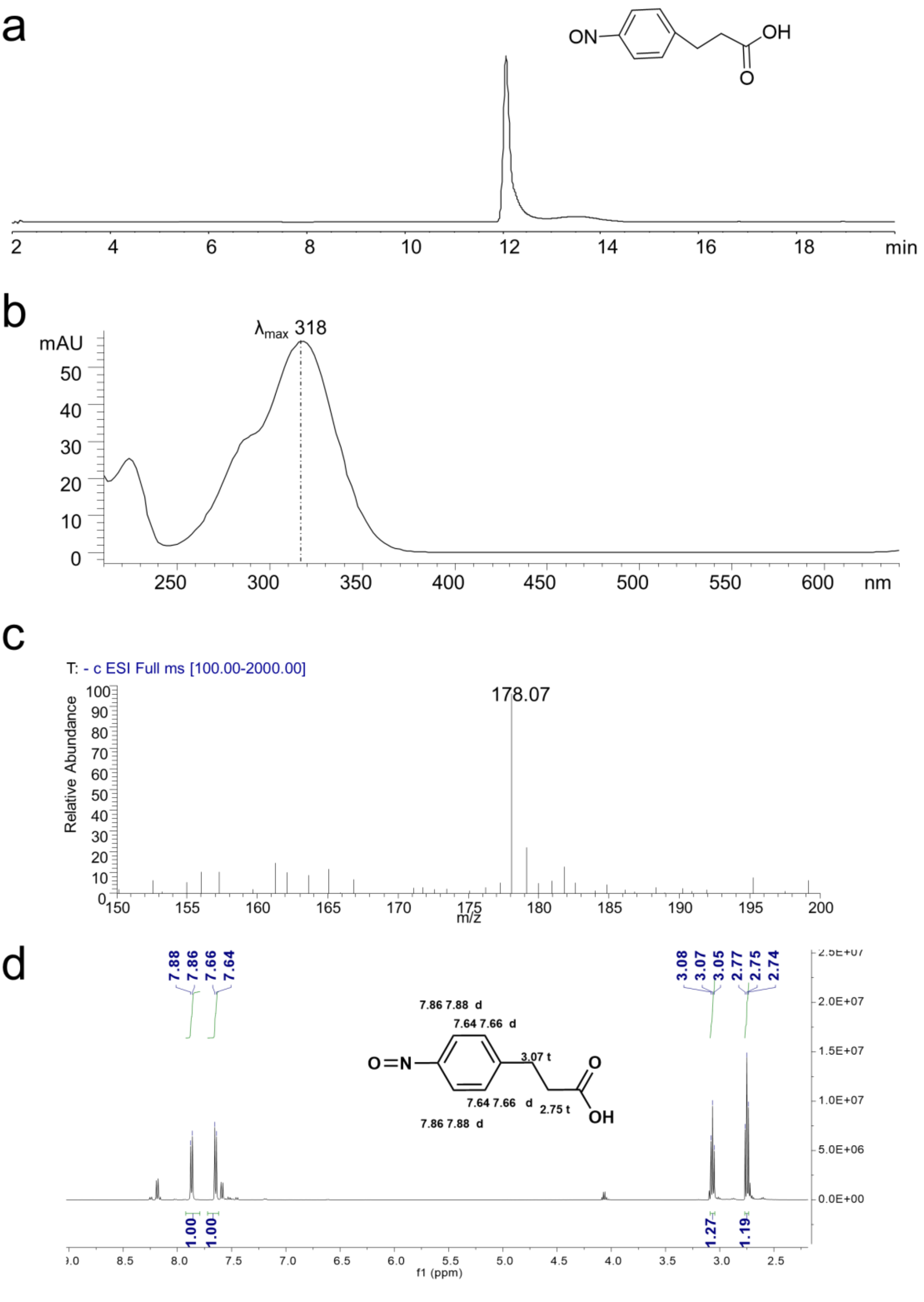

Supplementary Figure 25: Identification of synthesized *p*-nitroso phenypropionic acid. **a** HPLC analysis of *p*-nitroso phenypropionic acid at 370 nm. Retention time was 12.1 min. **b** UV/vis spectrum of *p*-nitroso phenypropionic acid. **c** MS of *p*-nitroso phenypropionic acid ([M-H]$^-$ 178.07). **d** ^{1}H NMR of synthesized *p*-nitroso phenypropionic acid in acetone-*d*6.(Note: nitroso arene is very active during the purification process and influence the NMR purity.)

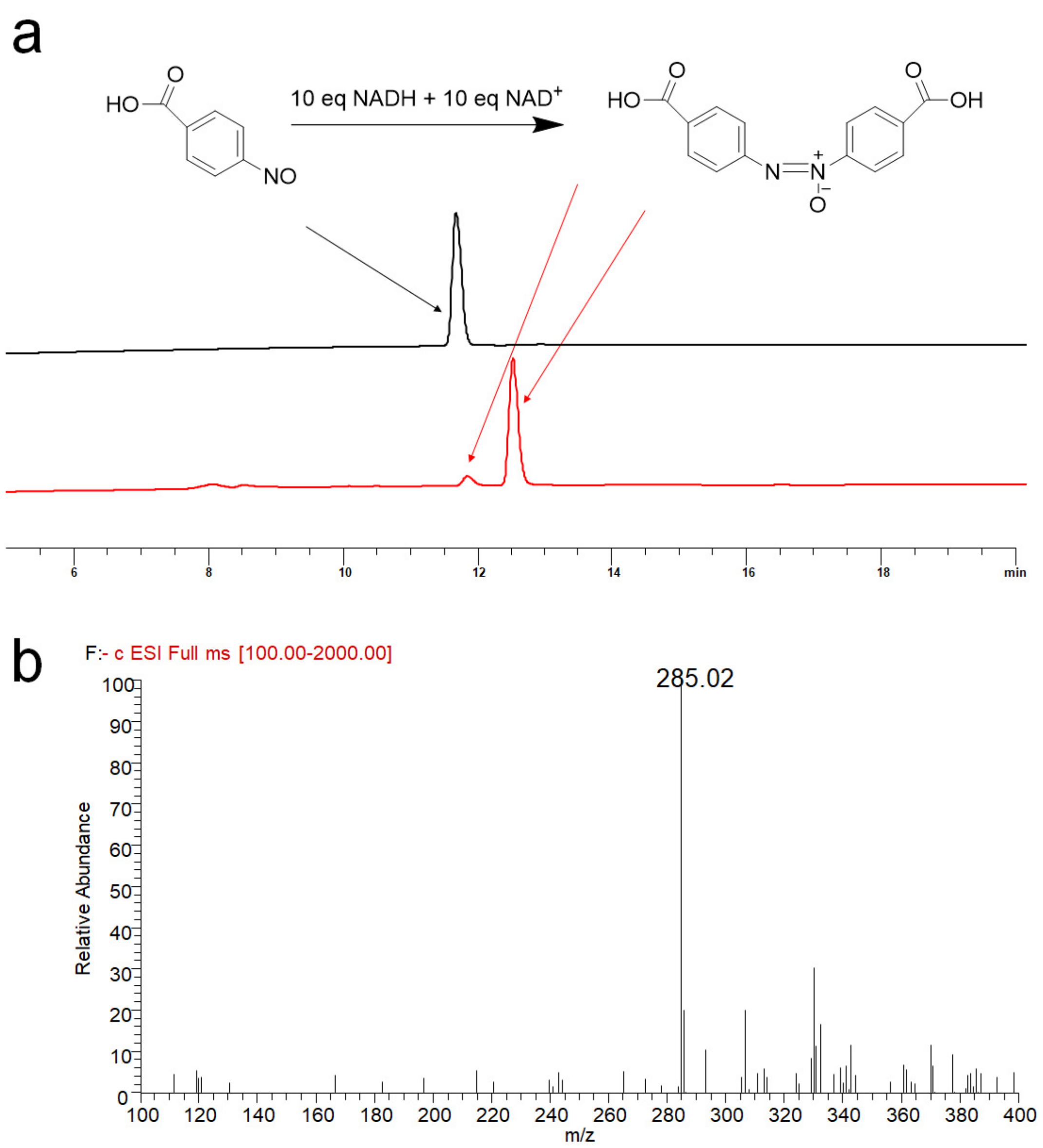

Supplementary Figure 26: LC-MS analysis of *p*-nitroso benzoic acid incubated with 10 eq NAD^+ and NADH for 1min. **a** HPLC analysis of standard *p*-nitroso benzoic acid (black) and *p*-nitroso benzoic acid incubated with 10 eq NAD^+ and 10 eq NADH for 1min (red) at 370 nm. No *p*-nitroso benzoic acid was detected according to HPLC (RT 11.13 min), indicating that all substrate was transformed to azoxy product (RT 13.01 min). **b** MS of the peak at 13.01 min ($[M-H]^-$ 285.02).

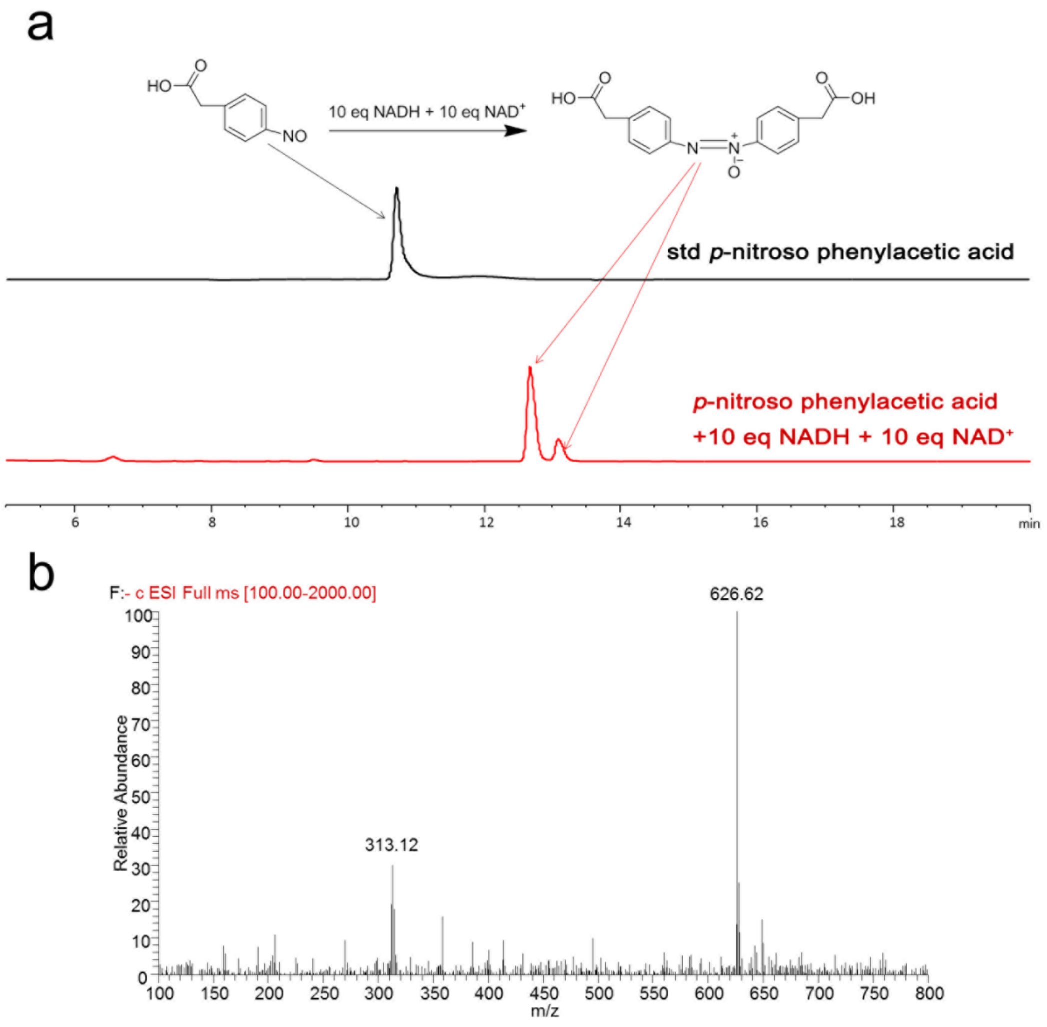

Supplementary Figure 27: LC-MS analysis of *p*-nitroso phenylacetic acid incubated with 10 eq NAD^+ and NADH for 1min. **a** HPLC analysis of *p*-nitroso phenylacetic acid (black) and *p*-nitroso phenylacetic acid incubated with 10 eq NAD^+ and 10 eq NADH for 1min (red) at 370 nm. Trace *p*-nitroso phenylacetic acid was detected according to HPLC (RT 10.76 min), indicating that most substrate was transformed to azoxy product (RT 12.7 min and RT 13.17 min). **b** MS of the peak at 12.7 min ($[M-H]^-$ 313.12, $[2M-H]^-$ 626.62).

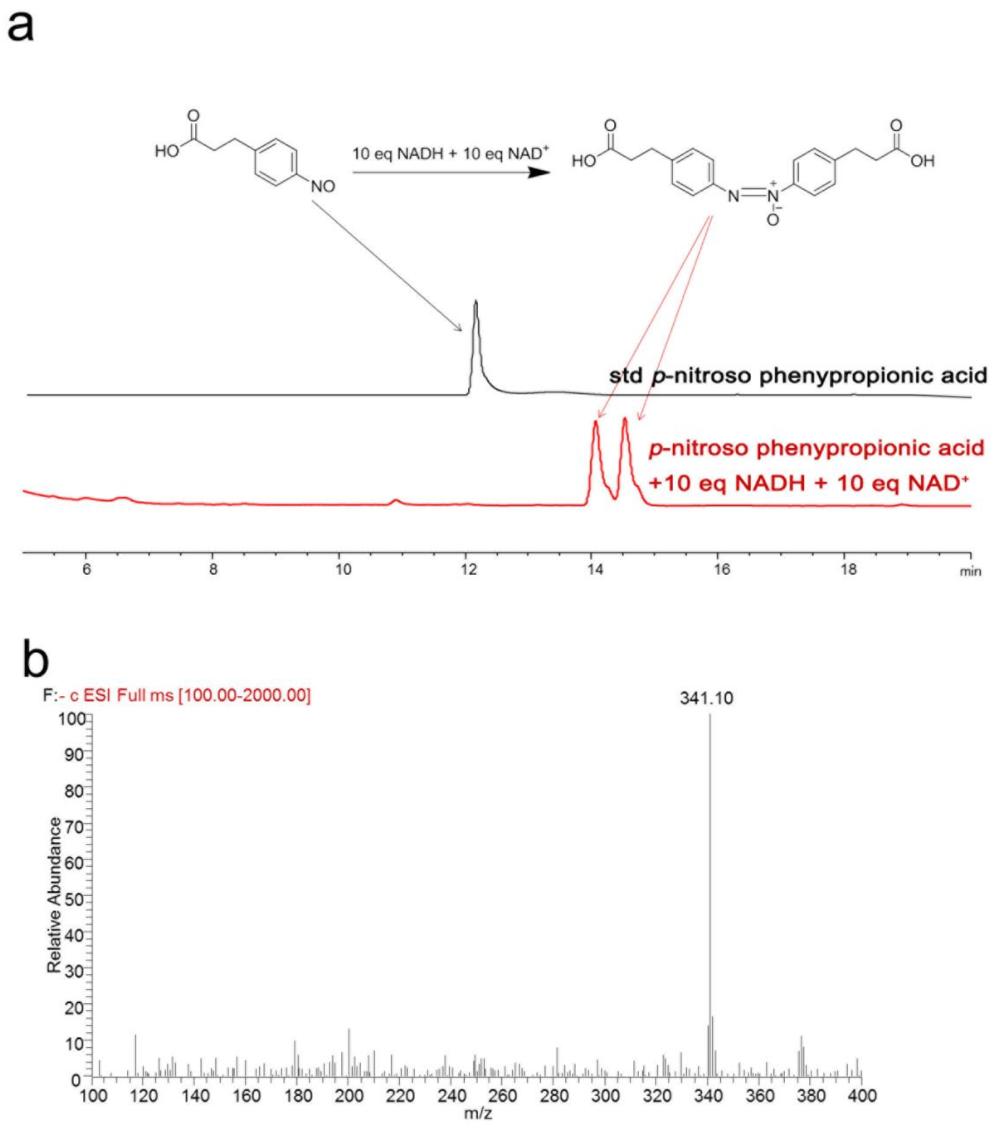

Supplementary Figure 28: LC-MS analysis of *p*-nitroso phenypropionic acid incubated with 10 eq NAD^+ and NADH for 1min. **a** HPLC analysis of *p*-nitroso phenypropionic acid (black) and *p*-nitroso phenypropionic acid incubated with 10 eq NAD^+ and 10 eq NADH for 1min (red) at 370 nm. Trace *p*-nitroso benzoic acid was detected according to HPLC (12.1 min), indicating that most substrate was transformed to azoxy product (14.3 min and 14.7). **b** MS of the peak at 14.3 min ($[M-H]^-$ 341.10).

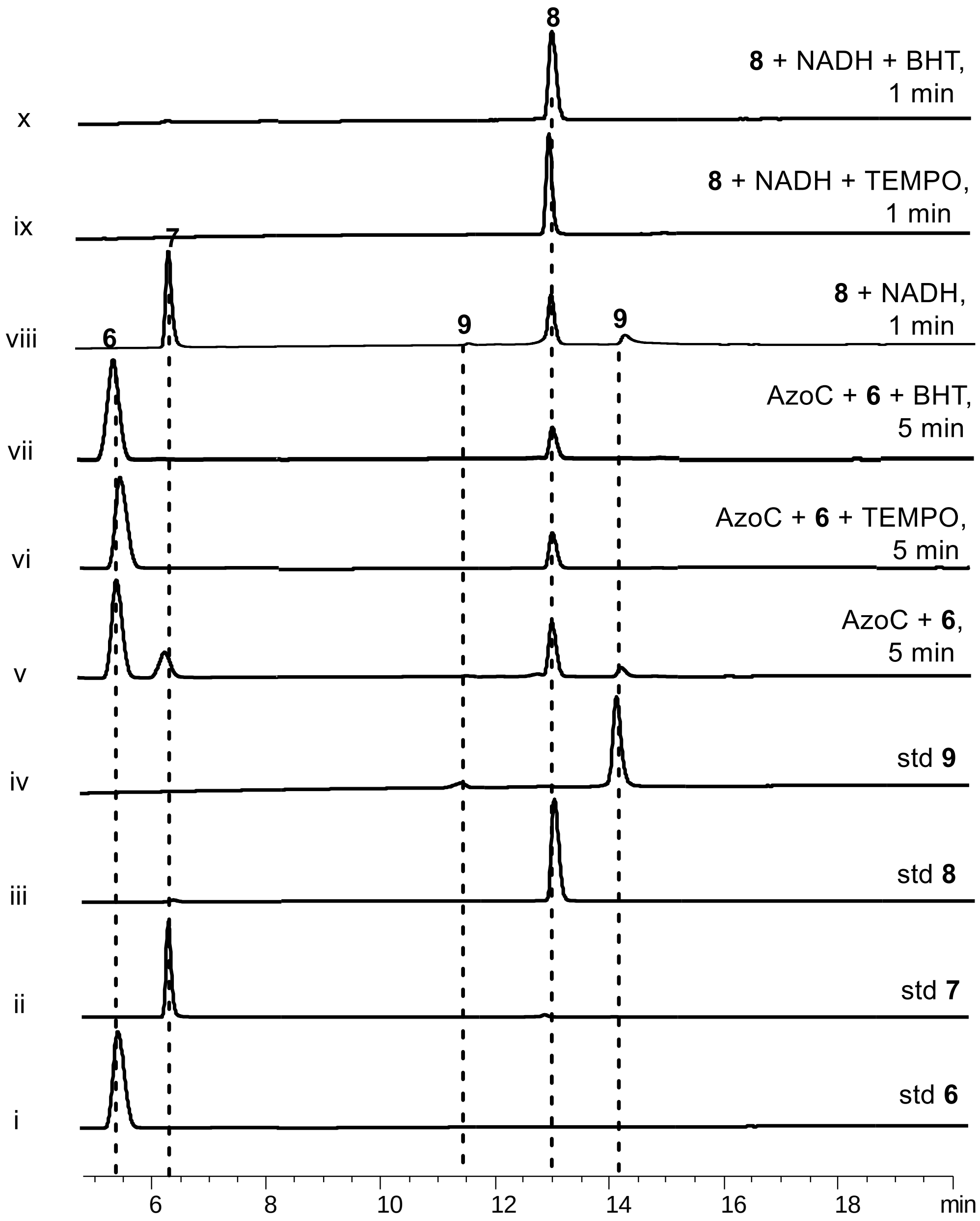

Supplementary Figure 29: HPLC analysis of AzoC's *in vitro* enzymatic reactions and non-enzymatic reactions with TEMPO and BHT at 345 nm. Reactions were performed in 20 mM HEPES buffer (pH 7). (i) standard **6**, (ii) standard **7**, (iii) standard **8**, (iv) standard **9**, (v) 100 μM **6** with 1 μM AzoC for 5 min, (vi) 100 μM **6** and 2 eq TEMPO with 1 μM AzoC for 5 min, (vii) 100 μM **6** and 2 eq BHT with 1 μM AzoC for 5 min, (viii) **8** with 10 eq NADH for 1 min, (ix) **8** with 10 eq NADH and 2 eq TEMPO for 1 min, (x) **8** with 10 eq NADH and 2 eq TEMPO for 1 min.

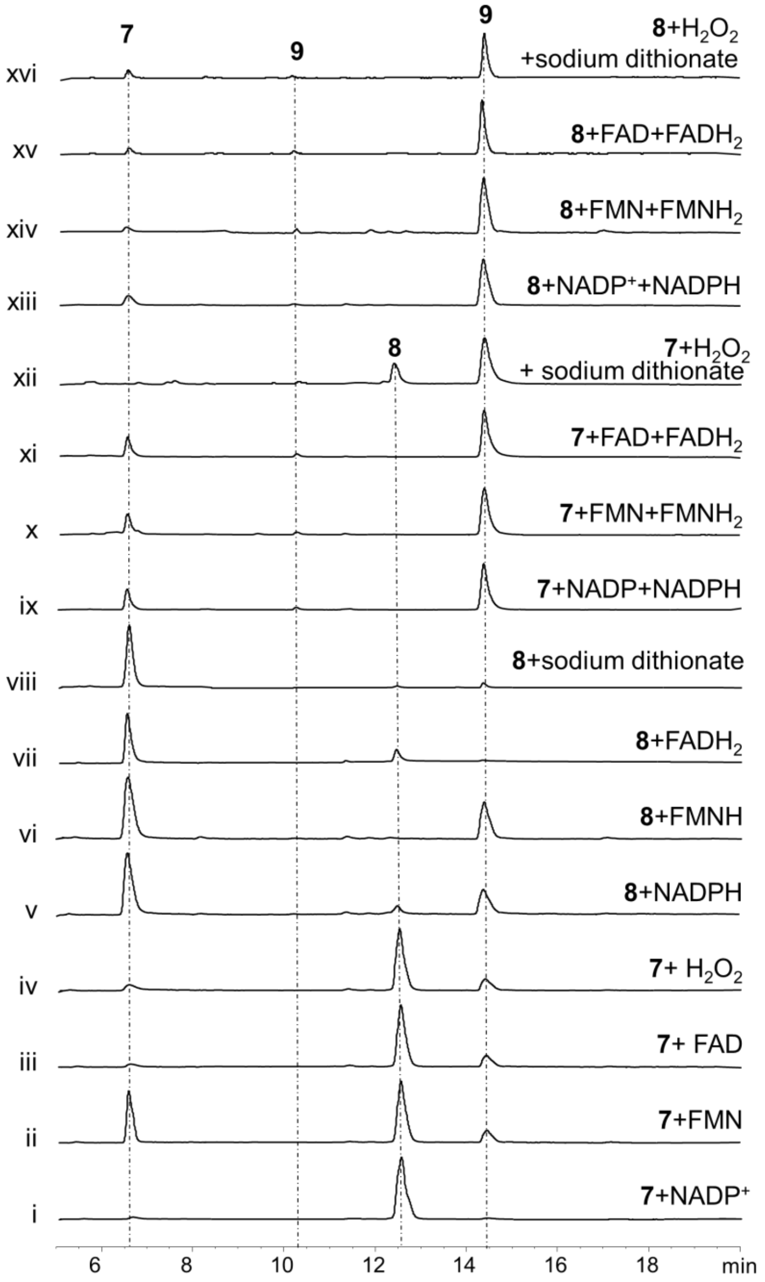

Supplementary Figure 30: HPLC analysis of **7** and **8**'s non-enzymatic *in vitro* reactions at 370 nm. Redox agents are $NADP^+$/ NADPH, FMN/$FMNH_2$, FAD/$FADH_2$, H_2O_2/sodium dithionate. Reactions were performed at 30 ℃ in 20 mM HEPES buffer (pH 7). (i) **7** with10 eq $NADP^+$ for 1 min, (ii) **7** with10 eq FMN for 1 min, (iii) **7** with 10 eq FAD for 1 min, (iv) **7** with 20 eq H_2O_2 for 1 min, (v) **8** with10 eq NADPH for 1 min, (vi) **8** with10 eq $FMNH_2$ for 1 min, (vii) **8** with10 eq $FADH_2$ for 1 min, (viii) **8** with 10 eq sodium dithionate for 1 min, (ix) **7** with10 eq NADPH and 10 eq $NADP^+$ for 1 min, (x) **7** with 10 eq $FMNH_2$ and 10 eq FMN for 1 min, (xi) **7** with 10 eq FAD and 10 eq $FADH_2$ for 1 min, (xii) **7** with 20 eq H_2O_2 and 10 eq sodium dithionate for 1 min, (xiii) **8** with10 eq NADPH and 10 eq $NADP^+$ for 1 min, (xiv) **8** with10 eq $FMNH_2$ and 10 eq FMN for 1 min, (xv) **8** with10 eq FAD and 10 eq $FADH_2$ for 1 min, (xvi) **8** with 20 eq H_2O_2 and 10 eq sodium dithionate for 1 min.

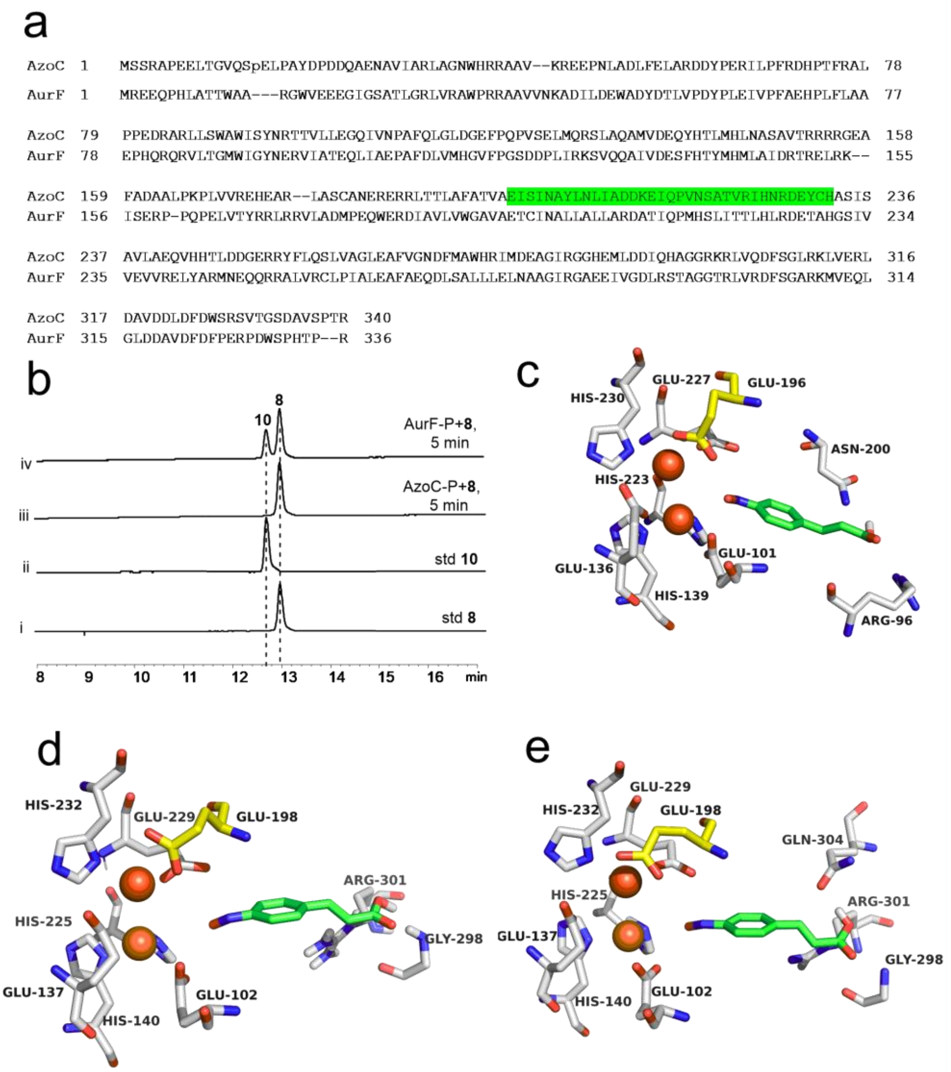

Supplementary Figure 31: Sequence alignment and *in silico* docking analysis of AurF, AzoC and AzoC-mutant. The models of AzoC are constructed with Modeler 9.18[10], and Gromacs 5.1.4[11]. The iron atoms are shown as brown spheres, and the Fe binding ligands of relative protein are surrounding the di-iron center and labeled. **a** Amino acid sequence alignment between AzoC and AurF. The sequence in AzoC colored in green was mutated to the consensus sequence in AurF. **b** HPLC analysis of AzoC-P and AurF-P' s *in vitro* biochemical reactions at 345 nm. Reactions were performed in 20 mM HEPES buffer (pH 7). (i) standard **8**, (ii) standard **10**, (iii) **8** with eq AzoC-P for 5 min, (iv) **8** with eq AurF-P for 5 min. **c** Docking of compound **8** into the active site of AurF. **d** Docking of compound **8** into the active site of AzoC. **e** Docking of compound **8** into the active site of AzoC-mutant.

Supplementary Tables.

Supplementary Table 1. Proposed gene function in azoxymycins biosynthetic gene cluster.

Protein	GenBank Accession Number	Predicted function
AzoA	KP687734	esterase
AzoB	KP687733	ketoreductase
AzoC	KP687735	*p*-aminobenzoate N-oxidase
AzoD	KP687736	EmrB/QacA subfamily drug resistance transporter
AzoE	KP687737	ABC substrate binding protein
AzoF	KP687738	ketosynthase
AzoG	KP687739	beta-ketoacyl synthase
AzoH	KP687740	3,4-AHBA carrier protein
AzoI	KP687741	KS I/II associated ACP
AzoO	KP687742	4′-phosphopantetheinyl transferase
AzoJ	KP687743	3,4-AHBA carboxyl group adenylation
AzoK	KP687744	*p*-aminobenzoate synthase
AzoL	KP687745	4-amino-4-deoxychorismate lyase
AzoM	KP687746	acyl dehydratase
AzoN	KP687747	acyl dehydratase

Supplementary Table 2: ^{1}H and ^{13}C-NMR assignment of synthesized **9**.

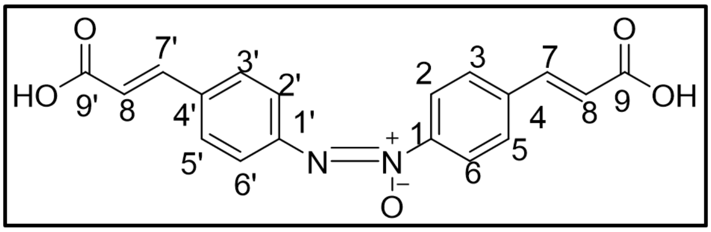

NO	^{1}H NMR	^{13}C NMR
1		148.01
1'		144.4
2	8.29,8.27d J = 8.8 Hz	122.68
2'	8.16,8.14 d J = 8.6 Hz	125.67
3	7.93,7.94 d J = 8.8 Hz	128.96
3'	7.86, 7.87 d J = 8.7 Hz	128.81
4		138.31
4'		135.81
5	7.93,7.94 d J = 8.8 Hz	128.96
5'	7.86, 7.87 d J = 8.7 Hz	128.81
6	8.29,8.27d J = 8.8 Hz	122.68
6'	8.16,8.14 d J = 8.6 Hz	125.67
7	7.64,7.67 d J = 16.0 Hz	141.32
7'	7.60, 7.63 d J = 15.9 Hz	142.22
8	6.7,6.72 d J = 16 Hz	123.06
8'	6.64,6.67 d J = 15.9 Hz	121.41
9		167.46
9'		163.85

Supplementary References:

1. Kobayashi, A. & Matsumoto, H. Studies on methylazoxymethanol, the aglycone of cycasin: Isolation, biological, and chemical properties. *Arch. Biochem. Biophys.* **110**, 373-380 (1965).
2. Blair, L.M. & Sperry, J. Natural products containing a nitrogen-nitrogen bond. *J. Nat. Prod.* **76**, 794-812 (2013).
3. Dembitsky, V.M., Gloriozova, T.A. & Poroikov, V.V. Pharmacological and Predicted Activities of Natural Azo Compounds. *Nat. Prod. Bioprospect.* (2017).
4. Fujiu, M. et al. Azoxybacilin, a novel antifungal agent produced by Bacillus cereus NR2991. Production, isolation and structure elucidation. *J. Antibiot (Tokyo).* **47**, 833-5 (1994).
5. Nakayama, M. et al. Novel antifungal antibiotics maniwamycins A and B. I. Taxonomy of the producing organism, fermentation, isolation, physico-chemical properties and biological properties. *J. Antibiot (Tokyo).* **42**, 1535-40 (1989).
6. Sugawara, A. et al. Jietacins, azoxy antibiotics with potent nematocidal activity: Design, synthesis, and biological evaluation against parasitic nematodes. *Eur. J. Med. Chem.* **145**, 524-538 (2017).
7. Alice, A.F., Lopez, C.S., Lowe, C.A., Ledesma, M.A. & Crosa, J.H. Genetic and transcriptional analysis of the siderophore malleobactin biosynthesis and transport genes in the human pathogen Burkholderia pseudomallei K96243. *J. Bacteriol.* **188**, 1551-1566 (2006).
8. Ye, Y., Aulinger, K., Arnold, N., Spahl, W. & Steglich, W. Biosynthesis of the azoxycarboxamide lyophyllin and formation of some of its unnatural analogues in fruit-bodies of Lyophyllum connatum. *Tetrahedron. Lett.* **38**, 8013-8016 (1997).
9. Kariya, Y., Kubota, T., Fromont, J. & Kobayashi, J. Pyrinadine A, a novel pyridine alkaloid with an azoxy moiety from sponge Cribrochalina sp. *Tetrahedron. Lett.* **47**, 997-998 (2006).
10. Webb, B. & Sali, A. Comparative Protein Structure Modeling Using MODELLER. *Curr. Protoc. Bioinformatics.* **54**, 5.6.1-5.6.37 (2016).
11. Abraham, M. et al. *GROMACS: High performance molecular simulations through multi-level parallelism from laptops to supercomputers*, (2015).
